# Interferon-γ in the tumor microenvironment promotes the expression of B7H4 in colorectal cancer cells, thereby inhibiting cytotoxic T cells

**DOI:** 10.1038/s41598-024-56681-3

**Published:** 2024-03-13

**Authors:** Zhi-liang Jing, Guang-long Liu, Na Zhou, Dong-yan Xu, Na Feng, Yan Lei, Li-li Ma, Min-shan Tang, Gui-hui Tong, Na Tang, Yong-jian Deng

**Affiliations:** 1 Department of Pathology, School of Basic Medical Sciences and Nan Fang Hospital, Southern Medical University, Guangzhou, 510515 China; 2 Guangdong Provincial Key Laboratory of Molecular Tumor Pathology, Guangzhou, 510515 China; 3 Department of Pathology, Affiliated Hospital of Guangdong Medical University, Zhanjiang, 524001 China; 4 Department of Pathology, Guangdong Provincial Hospital of Traditional Chinese Medicine, Guangzhou, 510120 China; 5 Department of Pathology, Dongguan Songshan Lake Tungwah Hospital, Dongguan, 523413 China; 6 Department of Pathology, Guangdong Provincial People’s Hospital (Guangdong Academy of Medical Sciences), Southern Medical University, Guangzhou, 510080 China; 7 Department of Pathology, The First Affiliated Hospital of Guangzhou Medical University, Guangzhou, 510415 China; 8 Department of Pathology, Shenzhen People’s Hospital (The Second Clinical Medical College, Jinan University, The First Affiliated Hospital, Southern University of Science and Technology), Shenzhen, 518020 China

**Keywords:** Colorectal cancer, Tumor microenvironment, IFN-γ, B7H4, CD8^+^ T cells, Immune cytotoxicity, Cancer, Immunology

## Abstract

The bioactivity of interferon-γ (IFN-γ) in cancer cells in the tumor microenvironment (TME) is not well understood in the current immunotherapy era. We found that IFN-γ has an immunosuppressive effect on colorectal cancer (CRC) cells. The tumor volume in immunocompetent mice was significantly increased after subcutaneous implantation of murine CRC cells followed by IFN-γ stimulation, and RNA sequencing showed high expression of B7 homologous protein 4 (B7H4) in these tumors. B7H4 promotes CRC cell growth by inhibiting the release of granzyme B (GzmB) from CD8^+^ T cells and accelerating apoptosis in CD8^+^ T cells. Furthermore, interferon regulatory factor 1 (IRF1), which binds to the B7H4 promoter, is positively associated with IFN-γ stimulation-induced expression of B7H4. The clinical outcome of patients with CRC was negatively related to the high expression of B7H4 in cancer cells or low expression of CD8 in the microenvironment. Therefore, B7H4 is a biomarker of poor prognosis in CRC patients, and interference with the IFN-γ/IRF1/B7H4 axis might be a novel immunotherapeutic method to restore the cytotoxic killing of CRC cells.

## Introduction

Interferon-γ (IFN-γ), also called immune interferon, is an important cytokine present in the tumor microenvironment (TME). IFN-γ is mainly induced by mitogens or cytokines (such as IL-12 and IL-18), and is expressed in immune cells, especially T cells and natural killer (NK) cells^1,2^. IFN-γ binds to its receptors on the cell membrane, including the two subunits IFNGR1 and IFNGR2, forming a receptor complex that in turn activates the downstream JAK-STAT1 signal transduction pathway^3^ and performs a series of biological functions.

Upon its initial discovery, IFN-γ was identified as an antiviral molecule^4^, but it differs from type I interferons (IFN-α and IFN-β). In addition to playing a role in the defense against infection, IFN-γ also plays a prominent role in tumor diseases. Studies have shown that in mouse models of lung and breast cancer, virotherapy with an IFN-γ-encoding oncolytic virus slows tumor growth, prolongs the survival time of tumor-bearing mice, and elicits effective antitumor responses^5,6^. In a phase II clinical trial for cutaneous T-cell lymphoma, the clinical application of a replication-deficient adenovirus encoding human IFN-γ elicited beneficial antitumor responses in most patients^7^. However, IFN-γ does not always have antitumor effects and some studies have suggested that it can promote tumor growth. IFN-γ can activate the immune activity of tumor cells and induce CXCL11 secretion in tumor cells, monocytes, endothelial cells, and fibroblasts. CXCL11 binds to CXCR7 and promotes angiogenesis and tumor growth^8^.

Colorectal cancer (CRC) is a solid malignancy with high morbidity and mortality worldwide^9^. Treatment options for CRC include surgery, radiotherapy, chemotherapy, and targeted therapy. The latest breakthrough in cancer treatment is immune checkpoint blockade (ICB) therapy, which involves blocking the binding of inhibitory receptors expressed on effector T cells in the TME to surface ligands on tumor cells^10^. Currently, the most widely used targets for tumor ICB therapy are cytotoxic T lymphocyte-associated protein 4 (CTLA-4) and programmed cell death protein 1 (PD-1) and programmed cell death protein ligand 1 (PD-L1)^11,12^.

IFN-γ is an early cytokine used in tumor immunotherapy; however, its effects on CRC cells remain unclear. Our study revealed a significant difference in the growth curves of subcutaneous tumors between immunodeficient BALB/c nude mice (IM-d-mice) and immunocompetent BALB/c mice (IM-c-mice) after IFN-γ treatment. In this study, we focused on exploring the effect of IFN-γ on the biological behavior of CRC, immunoregulatory mechanism of IFN-γ, and identification of potential immunotherapy targets.

## Materials and methods

### Patient tissues

CRC tumor tissues were obtained from 249 patients with complete follow-up data (11 years; Nan fang Hospital of Southern Medical University, Guangzhou, China). Patients or their relatives approved the use of these clinical materials for research purposes according to the Ethics Committee of Southern Medical University. The collected CRC tissue specimen were dehydrated, embedded in paraffin blocks, and subjected to hematoxylin–eosin and immunohistochemical (IHC) staining.

### Cell culture

The human-derived CRC cell lines SW480, DLD-1, HCT116, HT29, SW620, and LoVo were obtained from the American Type Culture Collection (ATCC; Manassas, VA, USA), and the mouse-derived colon cancer cell line CT26.WT was purchased from the Shanghai Chinese Academy of Sciences Cell Bank. All cell lines were cultured in RPMI-1640 medium (Biological Industries, Israel) supplemented with 10% fetal bovine serum (FBS; Gibco, USA), 100 U/ml penicillin, and 100 mg/ml streptomycin (catalog no. 15140122; Thermo Fisher Scientific). Cells were grown at 37 °C in a humidified atmosphere containing 5% CO_2_. All the cell lines were tested for *Mycoplasma* contamination in 2017. Recombinant cytokines IFN-γ (mouse), IFNα-2a (human), IFN-β (human), and IFN-γ (human) were purchased from GenScript Biotechnology Co., Ltd. (USA).

### Mice and treatments

Animal experiments were reviewed and approved by the Institutional Animal Care and Use Committee of our university. Four-week-old male immunocompetent BALB/c and immunode-ficient BALB/c nude mice (4 weeks old) were purchased from and maintained at the Central Laboratory of Animal Science at the Southern Medical University. Mice were randomly divided into groups of six mice per group. Cell suspensions were prepared from in vitro-cultured CT26.WT and HCT116 cells. Male BALB/c mice were injected subcutaneously with 1 × 10^6^ CT26.WT tumor cells on the upper right dorsal surface, and each male nude mouse was injected with 5 × 10^6^ HCT116 tumor cells. Each mouse in the experimental group was injected with 100 µL IFN-γ (Genscript, Cat.No.: Z02916) at a concentration of 100 ng/ml via the tail vein every 48 h, while each mouse in the control group was injected with 100 µL PBS via the tail vein. The mice were monitored until the subcutaneous tumors grew to 1.5 cm a diameter.

### Cell counting kit-8 (CCK-8) assay

CT26.WT, SW480, and DLD-1 cells in logarithmic growth phase were uniformly seeded in 96-well culture plate (800–1000 cells per well). Five wells were established for each group, and the cells were cultured for 24, 48, 72, 96, and 120 h. Then, 10 μl of CCK-8 reagent (catalog no. 40203ES76; Yeasen) was added to each well and the plates were incubated for 2 h. Absorbance was measured at 450 nm using a microplate reader (Bio-TAK, USA). The proliferation curves were plotted.

### Flow cytometry (FC) and sample preparation

Allograft tumors were minced and incubated in a digestion buffer (2 mg/ml collagenase IV [catalog no. C8160-1g**;** Solarbio), 0.5 mg/ml hyaluronidase (catalog no. H8030**;** Solarbio), and 0.1 mg/ml DNase I (catalog no. D8071-25 mg; Solarbio) in DMEM (catalog no. 06-1055-57-1ACS; Biological Industries)] at 37 °C for 30 min on a rotating shaker. The digested cell suspensions were then filtered through a 40 µm cell strainer and the cells were pelleted and washed with FACS buffer (2.5% FBS in PBS). Cells in single-cell suspensions were stained with a fluorophore-conjugated primary antibody diluted in FACS buffer for 30 min on ice in the presence of 1% Fc blocking solution (catalog no. 553141; BD Pharmingen). For whole blood samples, after fluorescent antibody staining, Red Blood Cell Lysis Buffer (catalog no. 00-4300-54; eBioscience) was added and the samples were incubated for 15 min. To stain intracellular components (such as Foxp3), fixation and permeabilization were performed using the Transcription Factor Buffer Set (catalog no 562574; BD Pharmingen), and intracytoplasmic or nuclear staining was performed for 40–50 min at 4 °C. Fluorochrome-conjugated antibodies specific to the following proteins were used for flow cytometric analysis: CD4 (clone RM4-5, BD Pharmingen), CD25 (clone PC61, BioLegend), Foxp3 (clone R16-715, BD Pharmingen), B7-H4 (clone 188, eBioscience), CD8a (clone 53-6.7, eBioscience), granzyme B (clone NGZB, eBioscience), perforin (clone eBioOMAK-D, eBioscience) and CD3 (clone 17A2, eBioscience). Fixable Viability Stain 780 (catalog no 565388, BD Horizon) was used to distinguish between viable and nonviable cells. The prepared samples were analyzed using an LSRFortessa X-20 flow cytometer (BD Biosciences). Data were analyzed using the FlowJo V10 software (BD Biosciences).

### Isolation and activation of CD8^+^T cells

CD8^+^ T cells were isolated from spleen and lymph nodes of OT-II mice by the EasySep Biotin Positive Selection Kit (STEMCELL Technologies, 17665), and activated with plated-bound anti-CD3 (BioXCell, BE0003, 5 μg/ml) and anti-CD28(BioXCell, BE0061, 2 μg/ml) for 48 h. After that, CD8^+^ T cells were treated with recombinant human IL-2 (peprotech, 200-02, 100 U/ml).CD8^+^ T cells were co-cultured with CT26-B7H4-OVA for 24 h, and then cells were analysis by FC.

### IHC

Formalin-fixed, paraffin-embedded (FFPE) blocks were sliced into 4-µm-thick sections, and the sections were placed onto microscope slides, which were then heated in a pressure cooker with 0.1% citrate buffer for 5 min. Sections were treated with 3% hydrogen peroxide for 10 min at room temperature and blocked with goat serum for 30 min at room temperature in a humidified chamber. Sections were incubated with primary antibodies diluted in goat serum in a humidified chamber overnight at 4 °C. Anti-CD8α antibody (clone CAL38; catalog no. ab237723; Abcam), and Anti-B7H4 ([EPR23665-20], ab252438). The sections were then incubated with biotinylated secondary antibodies (REAL™ EnVision, Dako), according to the manufacturer’s instructions,and counterstained with hematoxylin.

The staining intensity and percentage of positive tumor cells were evaluated using B7H4 staining. The semi-quantitative scoring system for the cytoplasmic staining intensity was as follows:0 (−), 1 (+), 2 (++), or 3 (+++). The degree of staining was based on the percentage of positive tumor cells and scored as follows:0 (< 5% positive cells), 1 (6–25% positive cells), 2 (26–50% positive cells), 3 (51–75% positive cells), or 4 (> 75% positive cells). The final staining scores were defined as 0 (−), 1–4 (+), 5–8 (++), and 9–12 (+++). The final staining score was used to divide the samples into a low-expression group (– and +) and a high-expression group (++ and +++).

CD8 staining results: Five visual fields per section were randomly selected from the central parenchyma of the tumor to count CD8^+^ lymphocytes using a 20× objective lens. The average of five visual fields was calculated to represent this section. According to the quartile classification system, 249 tissue sections exhibited either high (expression higher than the median value) or low expression.

### Western blotting analysis

Tissues or cells were lysed in RIPA buffer (catalog no. KGP702; Keygentec) containing protease and phosphatase inhibitor cocktail (catalog no. P1045; Beyotime) and clarified by centrifugation (12,000×*g* for 30 min at 4 °C). Protein concentrations were measured using a Pierce BCA Protein Assay Kit (catalog no. KGP902, Keygentec). Protein lysates (30 μg) were subjected to western blotting and protein bands were visualized using a Tianneng imaging system. The following primary antibodies were used: anti-VTCN1 (B7H4) (Proteintech, catalog no. 12080-1-AP), anti-PD-L1/CD274 (Proteintech, catalog no. 66248-1-Ig), anti-IRF1 (Proteintech, catalog no. 11335-1-AP), anti-IFNGR1 (Proteintech, catalog no. 10808-1-AP), anti-IFNGR2 (Proteintech, catalog no. 10266-1-AP), anti-JAK2 (Proteintech, catalog no. 17670-1-AP), anti-STAT1 (Proteintech, catalog no. 10144-2-AP), anti-CD86 (Proteintech, catalog no. 26903-1-AP) and anti-GAPDH (Proteintech, catalog no. 60004-1-Ig). The secondary antibody used was goat anti-mouse IgG (H + L)-HRP (Ray Antibody Biotech, catalog no. RM3001) and goat anti-rabbit IgG (H + L)-HRP (Ray Antibody Biotech, catalog no. RM3002). Because multiple cell lines and target proteins need to be detected, and some proteins have similar molecular weights, the blots have to be cut off before hybridization with antibodies, and Western blotting has to be carried out several times.

### Quantitative real time polymerase chain reaction (qRT-PCR)

qRT-PCR analysis was performed in triplicate using a SYBR^®^ Green Premix Pro Taq HS qPCR Kit (Accurate Biology, AG11701) and a 7500 Real-Time PCR System (Thermo Fisher Scientific). Expression data were normalized to V-set domain-containing T-cell activation inhibitor-1 (*Vtcn1*) expression using the 2^−△△Ct^ method. Primers were sourced from Sangon Biotech, and the sequences were as follows: *mVtcn1*(forward primer, TTCAAAGAAGGCAAAGATGAGC; reverse primer, GTCTCTGAGCTGGCATTATAGT) and *mGapdh* (forward primer,TGACTTCAACAGCGACACCCA; reverse primer, CACCCTGTTGCTGTAGCCAAA).

### Chromatin immunoprecipitation-qPCR (ChIP-qPCR)

Briefly, SW480 and DLD-1 cells (2 × 10^7^) were subjected to crosslinking, lysis, and sonication according to the manufacturer's instructions for the ChIP-IT^®^ Express Magnetic Chromatin Immunoprecipitation Kit and Shearing Kit (Active Motif, catalog no. 53008 and 53032). An anti-IRF-1 antibody (3 μg) was used to immunoprecipitate DNA fragments corresponding to the B7H4 promoter, which were amplified by qPCR using the following primers: B7H4-mut1:forward, TTGTTGCCCATCGTGGAG; reverse, GCAGCATACTGAGACTTCGTC. B7H4-mut2:forward, GCATAGATTCCAGGGCATC; reverse, AGGGATAAATGAGAGGCGG.

### Dual-luciferase reporter gene assay

Overexpression plasmids for the transcription factor IRF1 and the pGL4.10-B7H4 promoter wt, pGL4.10-B7H4 promoter mut1, and pGL4.10-B7H4 promoter mut2 vectors were constructed by Guangzhou Jidan Biotechnology Co., Ltd. First, well-grown SW480/DLD-1 cells were plated in a 24-well plate (5 × 10^4^ cells/well) and transfected with the corresponding plasmids for 48 h. Finally, the cells were subjected to luciferase assay using the Dual-Luciferase^®^ Reporter Assay System (Promega, catalog no. E1910) according to the manufacturer's instructions.

### Enzyme linked immunosorbent assay (ELISA)

Cytokine analysis of cell co-culture supernatants was performed in technical duplicates using the Human IFN-γ ELISA Kit (Elabscience, catalog no. E-EL-H0108c), and the Human IFN-β ELISA Kit (Elabscience, catalog no. E-EL-H0085c), according to the manufacturer's instructions. The absorbance values corresponding to cytokine levels were measured using a microplate reader (Bio-TAK, USA), and the concentrations were then calculated.

### Immunofluorescence assay

Tumor-associated fibroblasts(TAFs)were fixed with 4% paraformaldehyde, permeabilized with Triton X-100, blocked with goat serum, and incubated with an antibody recognizing fibroblasts (catalog no. CBL271), overnight at 4 °C. The cells were stained with a secondary antibody (1:100, Zhongshan Golden Bridge Biotechnology), the nuclei were stained with 4′,6-diamidino-2-phenylindole (DAPI; Sigma), and the cells were counted and examined using a confocal microscope (FV1000, Olympus).

### Statistical analysis

Each assay was performed in triplicate and independently repeated at least thrice. The results are expressed as mean ± SDs. Data were analyzed using Student’s *t* test or one-way analysis of variance (ANOVA) to determine statistical significance. Cell proliferation and subcutaneous tumor growth curves were analyzed using a two-way ANOVA. Pearson’s chi-square (χ^2^) test or Fisher’s exact test was used to analyze the relationship between B7H4/CD8 expression and clinicopathological features. Survival curves were generated using the Kaplan–Meier method. All analyses were two-sided and conducted using SPSS (version 17.0) or GraphPad Prism 7.00 software; values of P < 0.05) to indicate statistically significant differences.

### Ethics approval

This work was approved by the medical ethics committee of Nan fang Hospital, Southern Medical University, China.

### Feasibility and rationality

We confirm that all procedures were carried out in accordance with relevant guidelines and regulations. Animal experiments in our study were conducted according to ARRIVE 2.0 guidelines.

## Results

### IFN-γ stimulation of CRC cell proliferation depends on the immune microenvironment

**Figure 1:**
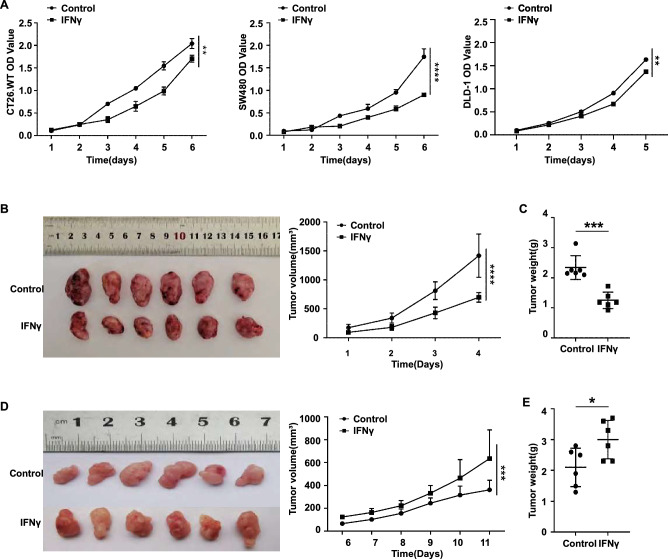
Effect of IFN-γ stimulation on the proliferation of CRC cells. (**A**) Evaluation of CRC cell proliferation by CCK-8 assays in vitro. Two-way ANOVA (**P < 0.01; ****P < 0.0001). (**B**) Volume curve and weight curve (**C**) of CT26 subcutaneous tumors in IM-d-mice. Two-way ANOVA (****P < 0.0001) (**B**), Student’s* t* test (***P < 0.001) (**C**). (**D**) Volume curve and weight curve (**E**) of CT26 subcutaneous tumors in IM-c-mice. Two-way ANOVA (***P < 0.001) (**D**), Student’s *t* test (*P < 0.05) (**E**).

We confirmed the effect of IFN-γ on the proliferation of CRC cells, both in vitro and in vivo. In our in vitro study, we evaluated the proliferation of three CRC cell lines, CT26.WT, DLD-1, and SW480, before and after IFN-γ stimulation using Cell Counting Kit-8 (CCK-8) assays and found that the absorbance value showed a decreasing trend in the IFN-γ stimulation group compared with the control group (without cytokine stimulation). These results showed that IFN-γ stimulation inhibited tumor cell proliferation in vitro (Fig. 1A).

In our in vivo study, we selected IM-d-mice and IM-c-mice for tumor growth assays using implanted CRC cells. First, we examined the growth trend of subcutaneously implanted CT26.WT tumors in IM-d mice. The IFN-γ stimulation group was compared with the control group, and the curves of both tumor volume (Fig. 1B) and final tumor weight (Fig. 1C) showed a decreasing trend in the IFN-γ stimulation group. These results suggest that IFN-γ stimulation can inhibit tumor growth in IM-d-mice, which is consistent with the results of the in vitro CCK-8 assay. However, in IM-c-mice, the tumors in IFN-γ-stimulated mice grew faster, and the final tumor volume (Fig. 1D) and weight were larger (Fig. 1E) than those in control mice. These results showed that IFN-γ stimulation promoted CRC tumor growth in IM-c mice. Therefore, we speculated that IFN-γ exerts an immunomodulatory effect through the TME.

### IFN-γ stimulation induces immunosuppression in CRC

**Figure 2:**
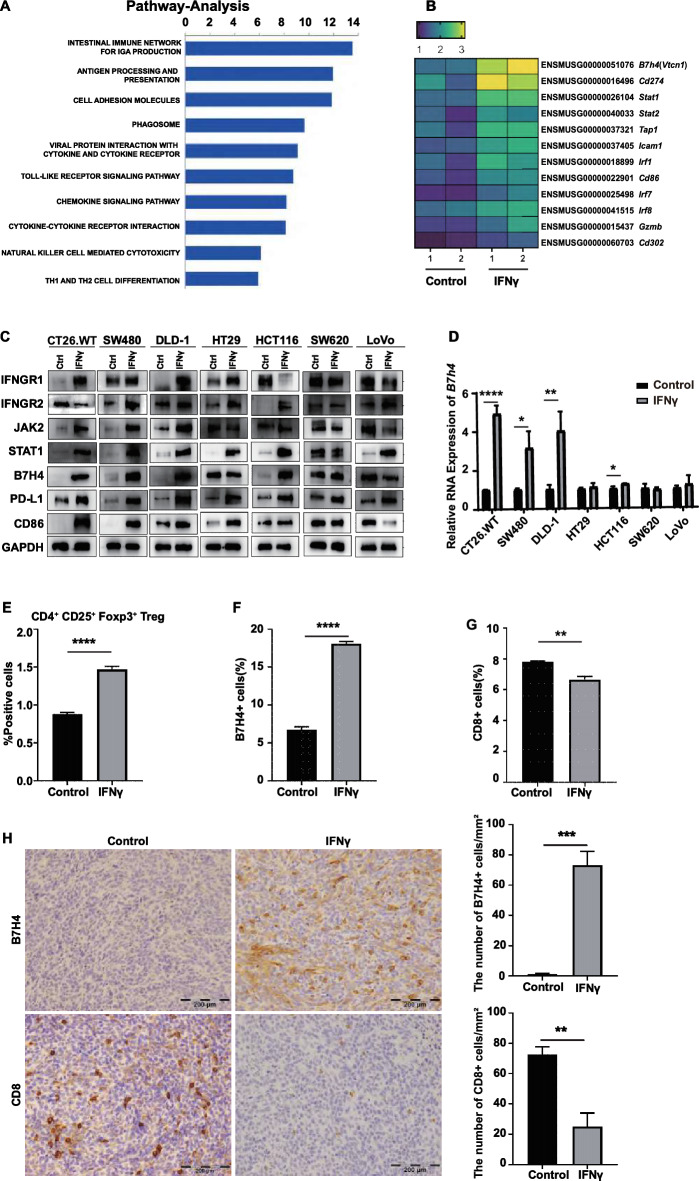
IFN-γ stimulation induces immunosuppression in CRC. (**A**) RNA-seq data of CT26.WT subcutaneous tumors showing the top ten pathways activated in the IFN-γ group (n = 2) compared with the control group (n = 3). The graph shows the category scores as -log10 (p values) determined by Fisher’s exact test. (**B**) RNA-seq data of CT26.WT subcutaneous tumors showing the top ten upregulated genes in the IFN-γ group (n = 2) compared with the control group (n = 2). The graph shows the category scores as -log10 (p values) determined by Fisher’s exact test. (**C**) The expression of interferon signaling pathway proteins in CRC cell lines was measured by Western blotting before and after IFN-γ stimulation. (The original images of Western blotting can be found in supplementary Figs. 3–9). (**D**) Comparison of relative *B7h4* RNA expression before and after IFN-γ stimulation in CRC cells, as determined by real-time qPCR. Multiple* t* tests. (**E**) T_reg_ cells in the peripheral blood of CT26.WT subcutaneous tumor-bearing IM-c-mice were analyzed by FC. (**F**) B7H4 protein expression in CT26.WT subcutaneous tumor cells was analyzed by FC. (**G**) The proportion of CD8^+^ T cells among CT26.WT subcutaneous tumor cells was determined by FC. (**H**) B7H4 and CD8 protein expression in CT26.WT subcutaneous tumors was detected by IHC staining. Scale bars, 200 μm. In (**E**) to (**H**), *P < 0.05, **P < 0.01, ***P < 0.001, and ****P < 0.0001 by Student’s* t* test.

We further explored the genetic alterations by which IFN-γ stimulation promoted the growth of subcutaneous tumors derived from implanted CT26.WT cells in IM-c-mice. Subcutaneous tumors were collected from the mice, and RNA sequencing (RNA-seq) was performed. In the subcutaneous tumor tissue of the IFN-γ-stimulated group, pathways related to suppression, antigen presentation, and cell adhesion molecules were significantly upregulated (Fig. 2A). The corresponding differentially expressed genes, *Gzmb, Cd274, Vtcn1, Irf1, Cd86* and *Stat1* were also significantly activated (Fig. 2B). Vtcn1, also known as B7H4, B7-H4, B7S1, and B7x, is a member of the B7 costimulatory protein family that was first identified in 2003. B7H4 can inhibit T cell activation, proliferation, and cytokine production^13,14^. The expression of the above-mentioned immunosuppression-related proteins was verified in cultured CRC cell lines in vitro, and the results of western blotting showed the upregulation of protein expression in CT26.WT, SW480, and DLD-1 cells (Fig. 2C). qRT-PCR confirmed that IFN-γ stimulation promoted the upregulation of B7H4 mRNA expression (Fig. 2D). However, B7H4 expression was not induced by IFN-γ exposure in several CRC cell line such as HT29, SW620 and LoVo. We believe that the possible reason is the difference of detection results due to the individual differences between cell lines and the heterogeneity of tumors.

FC and IHC analyses of mouse subcutaneous tumor tissues showed that in the IFN-γ stimulation group, the proportion of B7H4-expressing cells increased, while the proportion of CD8^+^ T cells infiltrating the tumor decreased (Fig. 2F–H). Peripheral blood samples were collected for FC. IFN-γ stimulation increased the proportion of regulatory T (T_reg_) cells in the peripheral blood of CT26 tumor-bearing IM-c mice (Fig. 2E). T_reg_ cells, a type of CD4^+^ T cells, specifically express the transcription factor FoxP3 in the nucleus and CTLA-4 and CD25 on their surface. T_reg_ cells are a subset of T cells that specifically mediate immunosuppression and play a role in maintaining immune self-tolerance and homeostasis^15^. In conclusion, we conclude that IFN-γ stimulation can induce immunesuppression of CRC. IFN-γ-induced elevated B7H4 and T_reg_ cells are synergistically involved in tumor immunosuppressive pathway. However, the immune regulatory factors in TME of CRC are complex, involving many immune cells and cytokines, such as CD8, CD4, NK, DC and MDSC cells, which may play a role in TME of CRC. In fact, it is necessary to detect immune cells in every direction in TME of CRC. This will be our next supplementary study.

### B7H4 promotes cell growth

To study the effect of B7H4 in the IFN-γ pathway on the growth of CRC cells, we first transfected B7H4 overexpression and interference vectors into CT26.WT cells to generate stable cell lines, and then verified these cell lines using western blotting and qRT-PCR (Supplementary Fig. 1).

**Figure 3:**
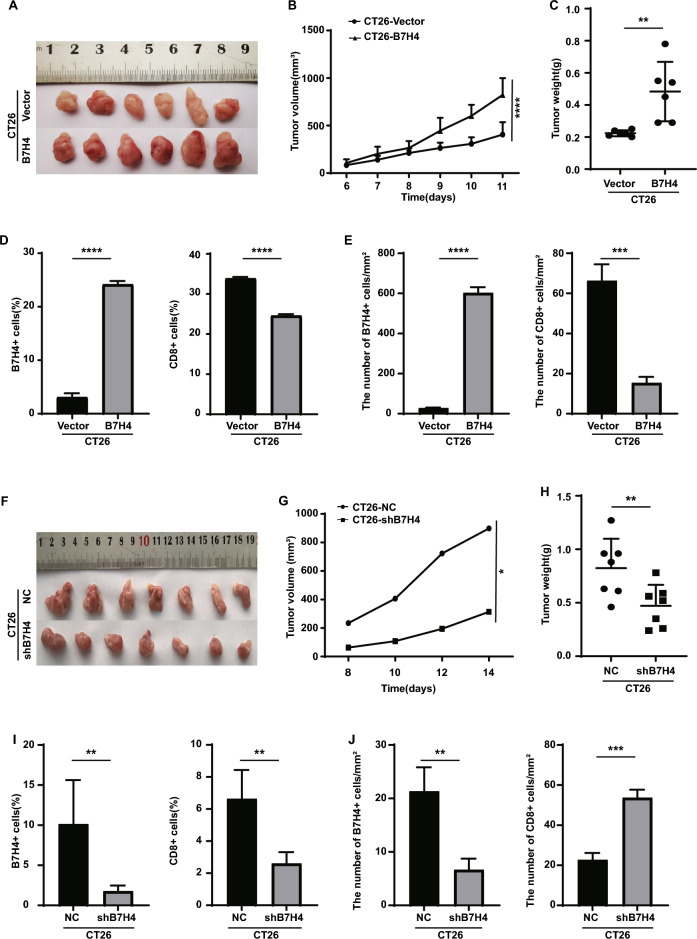
Effects of B7H4 on CRC tumor growth. (**A**) Subcutaneous tumors in the vector and CT26-B7H4 groups. (**B**) Subcutaneous tumor growth curves. Two-way ANOVA (****P < 0.0001). (**C**) Subcutaneous tumor weight. Student’s *t* test (**P < 0.01). (**D**) The proportions of B7H4- and CD8-positive cells in subcutaneous tumor tissue were determined by FC and (**E**) IHC staining. Student’s* t* test (***P < 0.001; ****P < 0.0001). (**F**) Subcutaneous tumors in the negative control (NC) and CT26-shB7H4 groups. (**G**) Growth curves of subcutaneous tumors in the NC and CT26-shB7H4 groups. Two-way ANOVA (*P < 0.05). (**H**) Weights of subcutaneous tumors in the NC and CT26-shB7H4 groups. Student’s *t* test (**P < 0.01). (**I**) The proportion of B7H4- and CD8-positive cells in subcutaneous tumor tissues in the NC and CT26-shB7H4 groups was determined by FC and (**J**) IHC staining. Student’s *t* test (**P < 0.01; ***P < 0.001).

The CT26.WT cell line with stable B7H4 overexpression (hereafter referred to as CT26-B7H4) was used for the subcutaneous tumorigenesis assay in IM-c-mice (Fig. 3A). The growth curve of the subcutaneous tumors (Fig. 3B) and statistical analysis of the final tumor weight (Fig. 3C) confirmed that B7H4 overexpression accelerated the growth of the CT26.WT subcutaneous tumors. Subcutaneous tumor tissue was subjected to FC (Fig. 3D) and IHC (Fig. 3E) analyses, which confirmed that the proportion of B7H4-positive cells increased and that of infiltrating CD8^+^ T cells (cytotoxic T lymphocytes, CTLs) decreased in the subcutaneous tumor tissue of B7H4-overexpressing mice. Simultaneously, we used the CT26.WT cell line with stable B7H4 interference (CT26-shB7H4) in a subcutaneous tumorigenesis assay in IM-c-mice, and found that interference with B7H4 expression inhibited the growth of CT26 subcutaneous tumors (Fig. 3F–H). The results of subcutaneous tumorigenesis assays showed that in the subcutaneous tumor tissue of mice in the B7H4 knockdown group, the proportion of B7H4-positive cells decreased (Fig. 3I,J), but the proportion of infiltrating CD8^+^ T cells increased (Fig. 3J). In view of the reverse trend of CD8 expression in CT26-shB7H4 tumors detected by FC (Fig. 3I) and IHC (Fig. 3J), we analyzed two possible reasons: (1) The weak staining of CD8 and the presence of necrotic cells in tumor cells interfere with the expression of CD8; (2) Due to the small number of CD8 T cells, the high threshold was adjusted in the process of setting the FC gate, resulting in the loss of CD8 cell statistical data.

Our research suggests that the expression of B7H4 in CRC has a trend opposite to that of the number of infiltrated CD8^+^ T cells, and that both of these factors control tumor progression. B7H4 inhibits the infiltration of CD8^+^ T cells into tumor tissues and plays an important role in promoting the growth of CRC cells.

### B7H4 inhibits CTLs

To verify the effect of B7H4 on immune activity and cytotoxicity (tumoricidal ability) of CD8^+^ T cells, we co-cultured isolated and activated CD8^+^ T cells with tumor cells from the CT26-B7H4 and CT26-shB7H4 groups. CD8^+^ T cell apoptosis and the release of cytotoxic mediators GzmB and perforin were detected using FC. Cytotoxic CD8^+^ T cells can utilize granzyme and perforin molecules packaged in cytotoxic granules to kill virus-infected tumor cells. Among the granzyme family members, GzmB has the strongest pro-apoptotic effects^16^.

**Figure 4:**
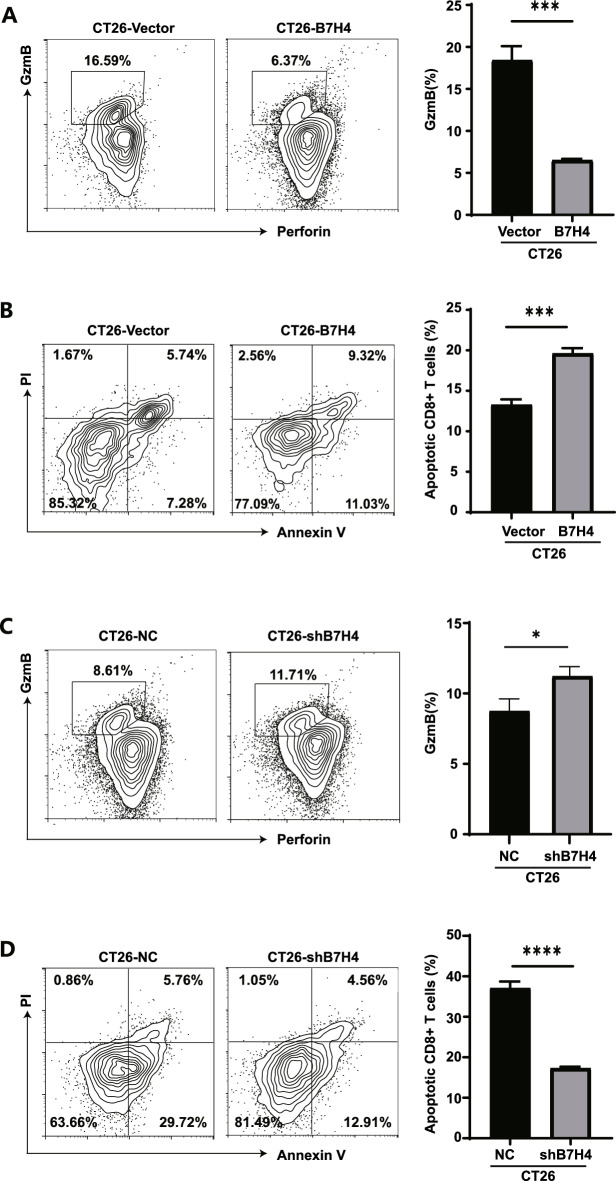
B7H4 inhibits CTLs. (**A**) Detection of cytotoxic cytokine release and (**B**) apoptosis of CD8^+^ T cells in the CT26-vector and CT26-B7H4 groups of co-cultured cells by FC. Student’s* t* test (***P < 0.001). (**C**) Detection of cytotoxic cytokine release and (**D**) apoptosis of CD8^+^ T cells in the CT26-vector and CT26-shB7H4 groups of co-cultured cells by FC. Student’s* t* test (*P < 0.05; ****P < 0.0001).

The proportion of CD8^+^ T cells that released the cytokine GzmB was significantly lower in the CT26-B7H4 co-culture group than that in the CT26-vector group (Fig. 4A). Moreover, apoptosis analysis showed that the apoptosis rate of CD8^+^ T cells in the peripheral blood was significantly increased in the CT26-B7H4 co-culture group (Fig. 4B). Conversely, the release of GzmB from CD8^+^ T cells in the CT26-shB7H4 co-culture group was higher than that in the CT26-vector group (Fig. 4C), whereas the apoptosis rate of CD8^+^ T cells significantly decreased in the CT26-shB7H4 co-culture group (Fig. 4D). These results confirmed that B7H4 exerts an immunosuppressive effect, reduces the cytotoxicity of CD8^+^ T cells, and weakens the tumoricidal ability of CD8^+^ T cells.

### IFN-γ-mediated regulation of B7H4 expression in CRC cells is related to IRF1

**Figure 5:**
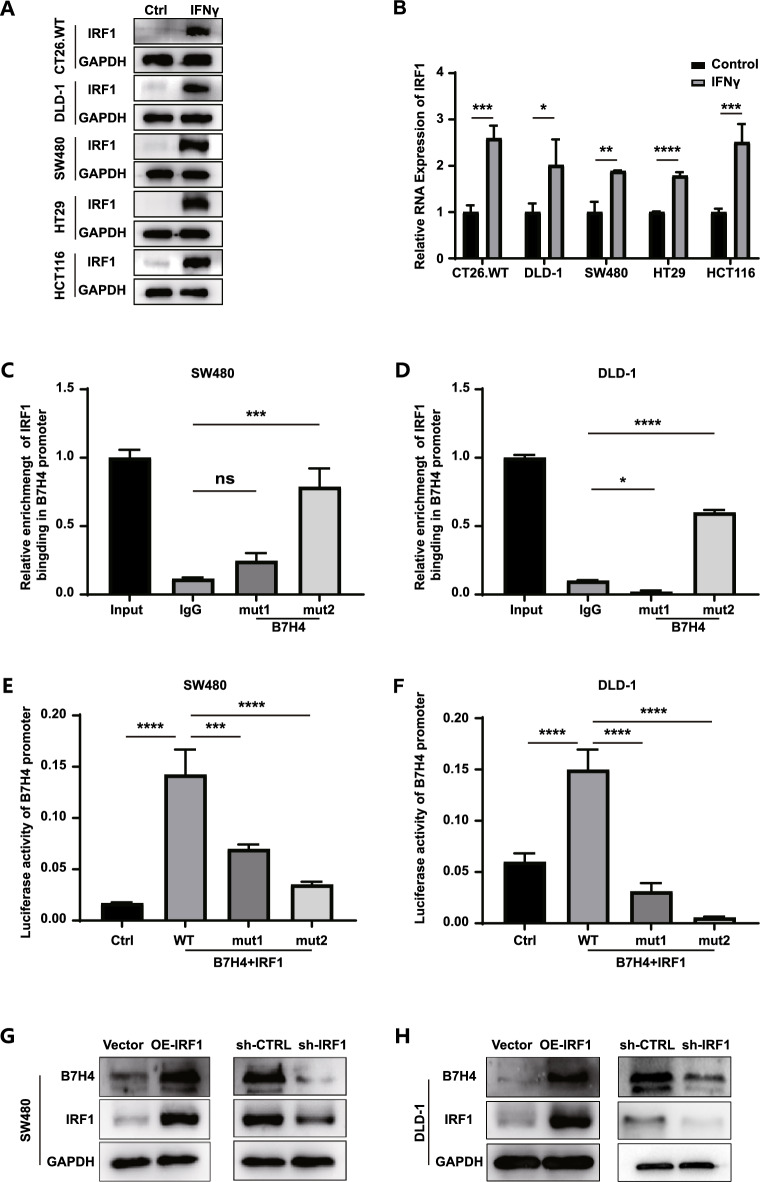
IRF1 regulates B7H4 expression. (**A**) The expression of IRF1 in CRC cells before and after IFN-γ stimulation was determined by Western blotting (the original images of Western blotting can be found in supplementary Figs. 10–14). (**B**) Representative qPCR results showing the expression of IRF1 before and after IFN-γ stimulation. (**C**) Relative enrichment of IRF1 binding in the B7H4 promoter, as determined by ChIP‒qPCR in SW480 and (**D**) DLD-1 cells. (**E**) Dual luciferase activity of the B7H4 promoter in SW480 and (**F**) DLD-1 cells. (**G**) Changes in B7H4 expression after overexpression of IRF1 (OE-IRF1) or silencing of IRF1 (sh-IRF1) were evaluated by Western blotting in SW480 and (**H**) DLD-1 cells. (The original images of Western blotting can be found in supplementary Figs. 15, 16). In (**B**), the data are presented as the means ± SDs. Student’s* t* test (*P < 0.05; **P < 0.01; ***P < 0.001; ****P < 0.0001). In (**C**) to (**F**), one-way ANOVA (*P < 0.05; **P < 0.01; ***P < 0.001; ****P < 0.0001).

To investigate the mechanism of B7H4 regulation, it is necessary to identify the transcription factor upstream of its target gene B7H4. Using the Gene-Cloud of Biotechnology Information (GCBI) database, we predicted that many transcription factors regulate B7H4. We selected the nuclear transcription factor IRF1, which is related to the interferon signaling pathway and had a higher score than the other predicted transcription factors. We then separately verified the expression of IRF1 in the interferon signaling pathway in CRC cell lines at the translational (Fig. 5A) and transcriptional levels (Fig. 5B). Both the protein and mRNA levels of IRF1 significantly increased after stimulation with IFN-γ. IRF1 is the first member of the interferon transcription factor family and was discovered^17^ in 1988. IRF1 is closely related to immune regulation: it inhibits the activity of dendritic cells^18^**,** positively regulates the development of CD8^+^ T cells^19^, and is a key gene in Th1 differentiation^20^.

We then determined the promoter sequence of the target gene *Vtcn1* (encoding B7H4) using the University of California, Santa Cruz (UCSC) database and predicted the binding site of the transcription factor IRF1 and the B7H4 promoter sequence using the JASPAR public database. The predicted results suggest that there are two possible binding sites (Supplementary Fig. 2). Therefore, we designed and synthesized qPCR primers based on these two putative binding sites and constructed knockout vectors containing the sequences of these binding sites. ChIP-qPCR and dual-luciferase reporter assays were used to verify the reliability of binding site predictions. Both experimental methods identified mut2, the sequence GGAAGGGAAAGC, located 824–835 bp upstream of the transcription start site of B7H4, as a more efficient and reliable binding site (Fig. 5C–F). Finally, we tested the effects of IRF1 overexpression and knockdown on the expression of the downstream protein, B7H4. Western blotting results showed that the transcription factor IRF1 positively regulated the expression of the downstream B7H4 protein (Fig. 5G,H).

### Relationship of B7H4 expression with clinicopathologic parameters in CRC patients

To clarify the clinical significance of B7H4 expression in CRC, we selected 249 CRC specimens from patients with complete follow-up data (11 years), prepared serial sections, and performed IHC staining.

**Figure 6:**
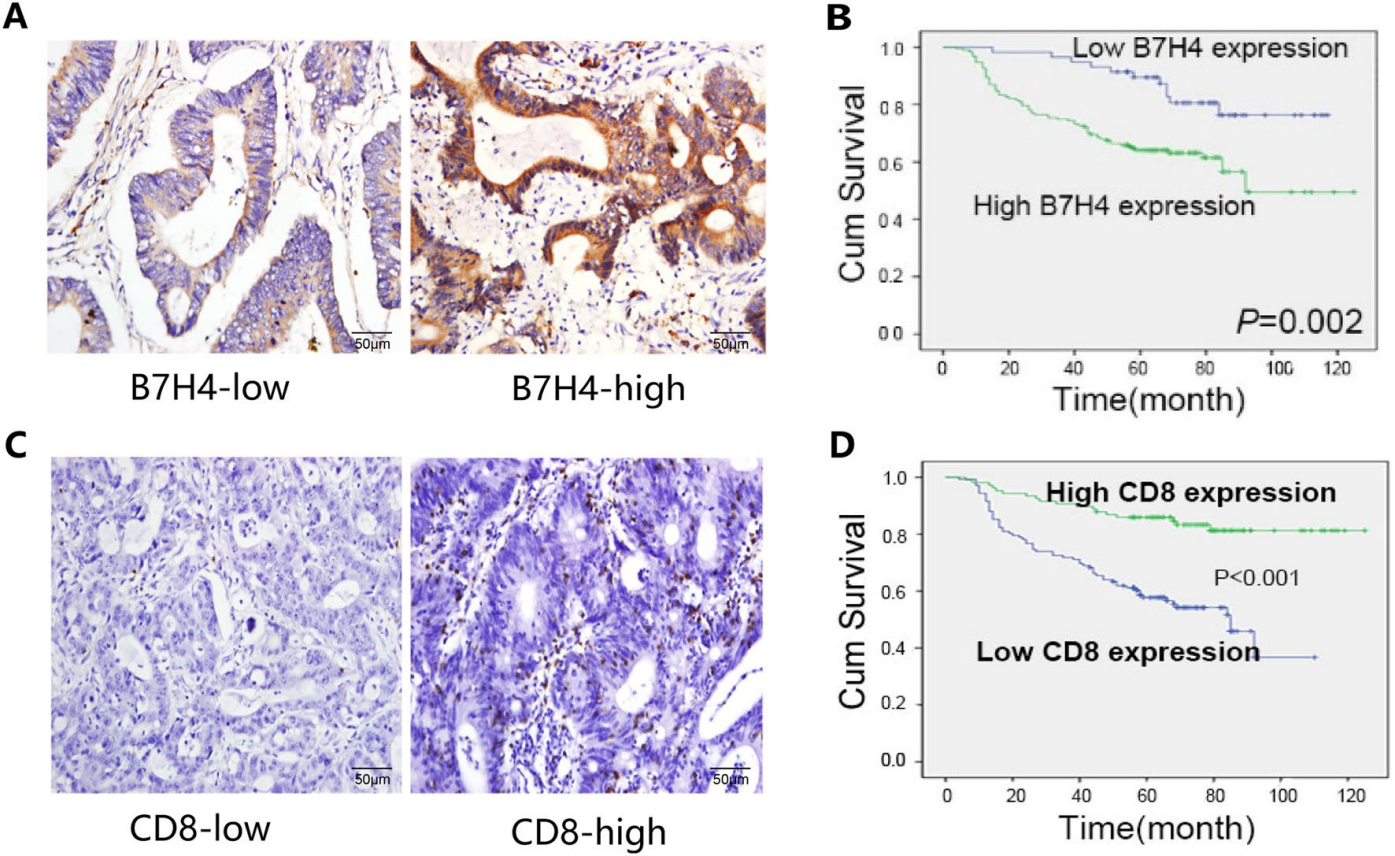
The expression and prognostic significance of B7H4 and CD8 in CRC tissues. (**A**) The expression of B7H4 in CRC tissues was evaluated by IHC staining. Scale bars, 50 μm. (**B**) The relationship between B7H4 expression and prognosis was confirmed by Kaplan–Meier survival analysis. (**C**) The expression of CD8 in CRC tissues was evaluated by IHC staining. Scale bars, 50 μm. (**D**) The relationship between CD8 expression and prognosis was confirmed by Kaplan–Meier survival analysis. Kaplan–Meier survival analysis (**P < 0.01; ****P < 0.0001).

B7H4 was localized mainly in the cytoplasm of tumor cells. The specimens were classified into two groups: one with high expression and the other with low B7H4 expression (Fig. 6A). Clinicopathological correlation analysis showed that high expression of B7H4 was positively correlated with advanced clinical stage, lymph node metastasis, and distant tumor metastasis (Table 1). The results of survival analysis showed that CRC patients with high B7H4 expression had a significantly shorter survival time and poorer prognosis (Fig. 6B). A positive correlation between high B7H4 expression and poor prognosis has also been identified in other solid malignancies, such as breast^21^ pancreatic^22^, non-small cell lung^23^, and ovarian cancers^24^. CD8 was mainly localized in the cell membranes of tumor-infiltrating lymphocytes in CRC samples (Fig. 6C), and the degree of CD8^+^ T-cell infiltration was mainly related to the degree of tumor differentiation and distant metastasis status (Table 2). The results of the survival analysis showed that the survival time of CRC patients with low CD8 expression was significantly shorter, and the prognosis was poorer (Fig. 6D).

**Table 1:** B7H4 expression status-dependent clinicopathologic features in CRC (n = 249).

Variables	N	B7H4-low	B7H4-high	*P*
Age				0.264
< 60 years	119	24	95	
≥ 60 years	130	34	96	
Gender				0.425
Male	152	38	114	
Female	97	20	77	
Tumor stage				0.001
I	52	21	31	
II	113	26	87	
III/IV	84	11	73	
Lymph node metastasis				0.007
Absence	165	47	118	
Presence	84	11	73	
Distant metastasis				0.015
M0	146	42	104	
M1	103	16	87	
Tumor location				0.995
Rectum	113	31	102	
Colon	116	27	89	
Differentiation				0.078
WD	105	33	72	
MD	111	20	91	
PD	19	3	16	
Mucinous/signet	14	2	12	
Size				0.503
< 4 cm	77	20	57	
≥ 4 cm	172	38	134

**Table 2:** CD8 expression status-dependent clinicopathologic features in CRC (n = 249).

Variables	N	CD8-low	CD8-high	*P*
Age				0.044
< 60 years	119	60	59	
≥ 60 years	130	82	48	
Gender				0.729
Male	152	88	64	
Female	97	54	43	
Tumor stage				0.090
I	52	23	29	
II	113	66	47	
III/IV	84	53	31	
Lymph node metastasis				0.168
Absence	165	89	76	
Presence	84	53	31	
Distant metastasis				0.000
M0	146	64	82	
M1	103	78	25	
Tumor location				0.323
Rectum	133	72	61	
Colon	116	70	46	
Differentiation				0.015
WD	105	52	53	
MD	111	70	41	
PD	19	8	11	
Mucinous/signet	14	12	2	
Size				0.596
< 4 cm	77	42	35	
≥ 4 cm	172	100	72	

WD, well differentiated; MD, moderately differentiated; PD, poorly differentiated. P-values were calculated using the chi-squared test or Fisher’s exact test.

### Source of interferon in the TME of CRC

The TME, the internal environment in which tumor cells exist, includes not only the tumor cells themselves but also other diverse cells and structures, such as microvessels, fibroblasts, immune and inflammatory cells in the surrounding intercellular milieu, and diverse cytokines secreted by these cells. Interferons are important cytokines in the TME. However, only certain cell types secrete IFN. Hematopoietic cells are the main cellular source of IFN-α, whereas fibroblasts are the main cellular source of IFN-β^25^. Initially, it was believed that only CD4^+^ T helper type 1 (Th1) lymphocytes, CD8^+^ cytotoxic T lymphocytes, and NK cells produce IFN-γ^26,27^.However, current evidence indicates that other cells such as B cells, natural killer T (NKT) cells and antigen-presenting cells (APCs) can also secrete IFN-γ^28–32^.

**Figure 7:**
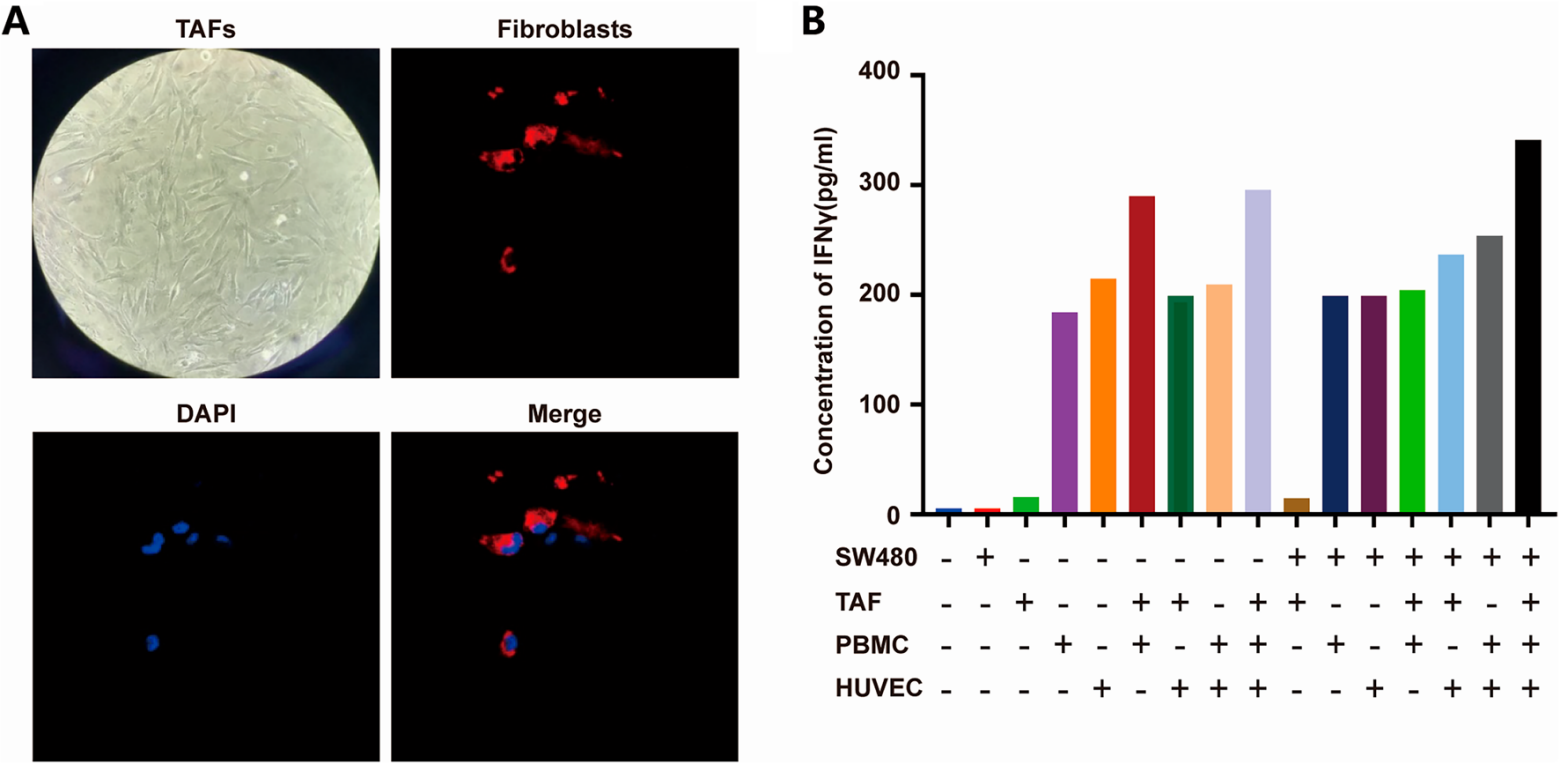
Source of IFN-γ in the TME of CRC. (**A**) Identification of TAFs by immunofluorescence staining and morphological analysis. (**B**) Quantification of IFN-γ secretion in the TME by ELISA.

To verify this hypothesis, we simulated the TME in vitro with a focus on SW480 CRC cells, selected primary isolated TAFs, and identified these cells using immunofluorescence staining (Fig. 7A). Peripheral blood mononuclear cells (PBMCs) and human umbilical vein endothelial cells (HUVECs) were co-cultured as non-tumor components to determine the source of IFN. The co-culture supernatant was subjected to solid-phase ELISA, and the results (Fig. 7B) showed that the IFN-γ level was obviously increased in the PBMC and HUVEC monoculture groups and in the PBMC and HUVEC co-culture groups but was very low in both the TAF and SW480 cell monoculture groups and in the TAF and SW480 cell co-culture groups.

Our study suggests that PBMCs and HUVECs in the TME are primary cellular sources of IFN-γ. However, more detailed research is needed to confirm the precise source of IFN-γ in the inflammatory cells.

## Discussion

Although IFN-γ has long been considered to exhibit antitumor activity, clinical trials have shown contradictory results regarding its effectiveness or ineffectiveness, preventing its clinical application. The main reason that IFN-γ cannot be applied clinically is its antitumor mechanism, the signaling pathway that it mediates, and the specific effect of its activity on the tumor immune environment is unclear. In our study, we found that although there is a balance between immune activation and immune-suppression in the TME, due to the existence of IFN-γ/IRF1/B7H4 regulatory axis seems to break this balance. Inflammatory cells in the TME secrete large amounts of IFN- γ, and then regulate the production of B7H4 by target cells to induce immunosuppressive effect.

The functional status of immune cells and cytokines in the TME partially determines the survival of tumor cells. The cytokine interleukin-1 α (IL-1 α) promotes the survival of breast cancer cells by inducing the expression of the key tumor survival-promoting cytokine, TSLP^33^. Cytokines in the TME facilitate tumorigenesis, progression, metastasis, and distant colonization of malignant cells in pancreatic ductal adenocarcinoma through their receptors^34^. The immunological effect of IFN-γ, an immunoactive cytokine, on carcinogenesis in TME is unclear. Our study found that the effect of IFN-γ on CRC cells varied with the immune microenvironment. IFN-γ exerted an inhibitory effect on CRC cells in IM-d-mice but promoted tumor growth in CRC cells in IM-c-mice. Because of the pleiotropic and complex effects of IFN-γ, its overall effect on tumor growth in the TME depends on the balance between antitumor and pro-tumor IFN-γ signaling.

To harness the antitumor bias of IFN-γ in tumor immunotherapy, it is necessary to understand the effector molecules involved in IFN-γ-induced immunosuppression. Previous studies have shown that IFN-γ promotes the expression of the inhibitory molecules PD-L1/PD-1, CTLA-4, indoleamine 2,3-dioxygenase 1 (IDO1) and inducible nitric oxide synthase (INOS)^35–38^, all of which limit antitumor immunity. Our study is the first to show that B7H4, another immunosuppressive molecule in the IFN-γ signaling pathway, plays a distinct role in CRC. The expression of B7H4 is regulated by the transcription factor IRF1, and induces an immunosuppressive effect by promoting the release of GzmB from CD8^+^ T cells and promoting apoptosis in CD8^+^ T cells. We also proved that simply knocking out B7H4 (i.e., the *Vtcn1* gene) can inhibit tumor growth. Therefore, immunotherapy targeting the IFN-γ/IRF1/B7H4 axis is promising.

Currently, strategies targeting CTLA-4 and PD-1 on CTLs and their main ligand PD-L1 on cancer cells have been tested in clinical trials^39,40^. B7H4 is expected to be another ICB target, after PD-L1/PD-1 and CTLA-4. Our clinical research results showed that the poor prognosis of patients with CRC is related to high expression of B7H4 or low infiltration levels of CTLs. Based on this finding, if an anti-B7H4 monoclonal antibody neutralizes the immunosuppressive effect and restores the cytotoxic effect of CTLs on CRC cells, CRC tumor progression may be altered. This will be the focus of future studies. B7H4 is also upregulated in a variety of tumors, including ovarian and breast cancer^41^. Dangaj's research in ovarian cancer showed that the use of recombinant antibodies to target B7H4 can restore the activation of the T-cell activation pathway^42^. Moreover, pharmacological inhibition of B7H4 glycosylation has been reported to restore antitumor immunity in immune-cold breast tumors^43^. These findings support our view that inhibiting B7H4 function and restoring T-cell function in patients with cancer has potential therapeutic value.

**Figure 8:**
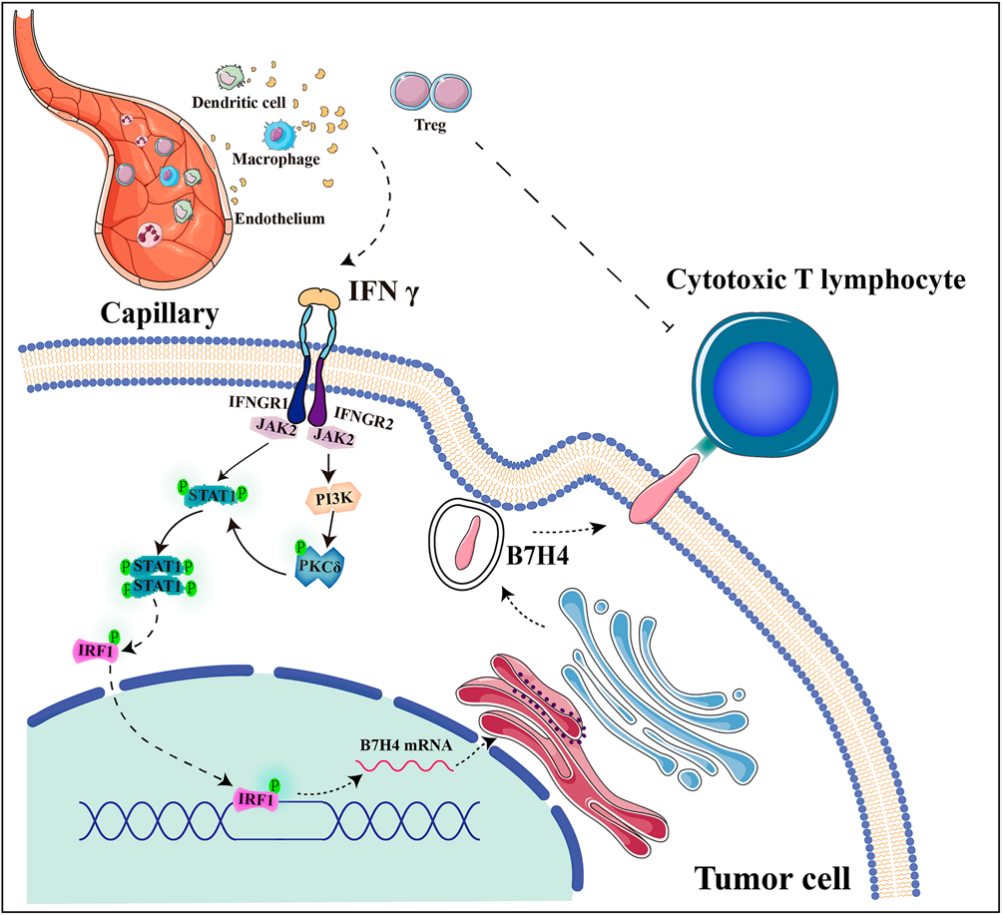
Graphical abstract. Diagram showing the regulatory pathway of IFN-γ and its effects on CRC cells in the TME.

In summary, the IFN-γ/IRF1/B7H4 axis may promote CRC progression by inhibiting immune response (Fig. 8). Thus, B7H4 can be used as a marker of poor prognosis in patients with CRC and may be a new target for CRC immunotherapy.

### Supplementary Information

Supplementary Information.

## Data Availability

The datasets generated in the current study can be obtained in the [GEO] repository, [series GSE252712].

## Abbreviations

IFN-γ: Interferon-γ
TME: Tumor microenvironment
CRC: Colorectal cancer
B7H4: B7 homologous protein 4
GzmB: Granzyme B
IRF1: Interferon regulatory factor 1
IM-d-mice: Immunodeficient BALB/c nude mice
IM-c-mice: Immunocompetent BALB/c mice
ICB: Immune checkpoint blockade
FC: Flow cytometry
IHC: Immunohistochemical
CTLA-4: Cytotoxic T lymphocyte-associated protein 4
PD-L1: Programmed cell death protein ligand 1
PD-1: Programmed cell death protein 1
CTLs: Cytotoxic T lymphocytes
