# Supplementary material for: Interferon-γ in the tumor microenvironment promotes the expression of B7H4 in colorectal cancer cells, thereby inhibiting cytotoxic T cells
Source: Sci Rep. 2024 Mar 13;14:6053. doi: 10.1038/s41598-024-56681-3 (PMC10937991; doi:10.1038/s41598-024-56681-3)
Supplement: Supplementary file 1 — Supplementary Information. [file 41598_2024_56681_MOESM1_ESM.docx]

**Supplemental Information**

**Interferon-γ in the tumor microenvironment promotes the expression of B7H4 in colorectal cancer cells, thereby inhibiting cytotoxic T cells**

**Zhi-liang Jing^1,2,3#^,** **Guang-long Liu^1,2#^ ,Na Zhou^4#^, Dong-yan Xu^1,2#^, Na Feng^5^ , Yan Lei^1,2^, Li-li Ma^6^, Min-shan Tang^1,2^ , Gui-hui Tong^7^, Na Tang^8^** **^✉^ , Yong-jian Deng^1,2✉^**


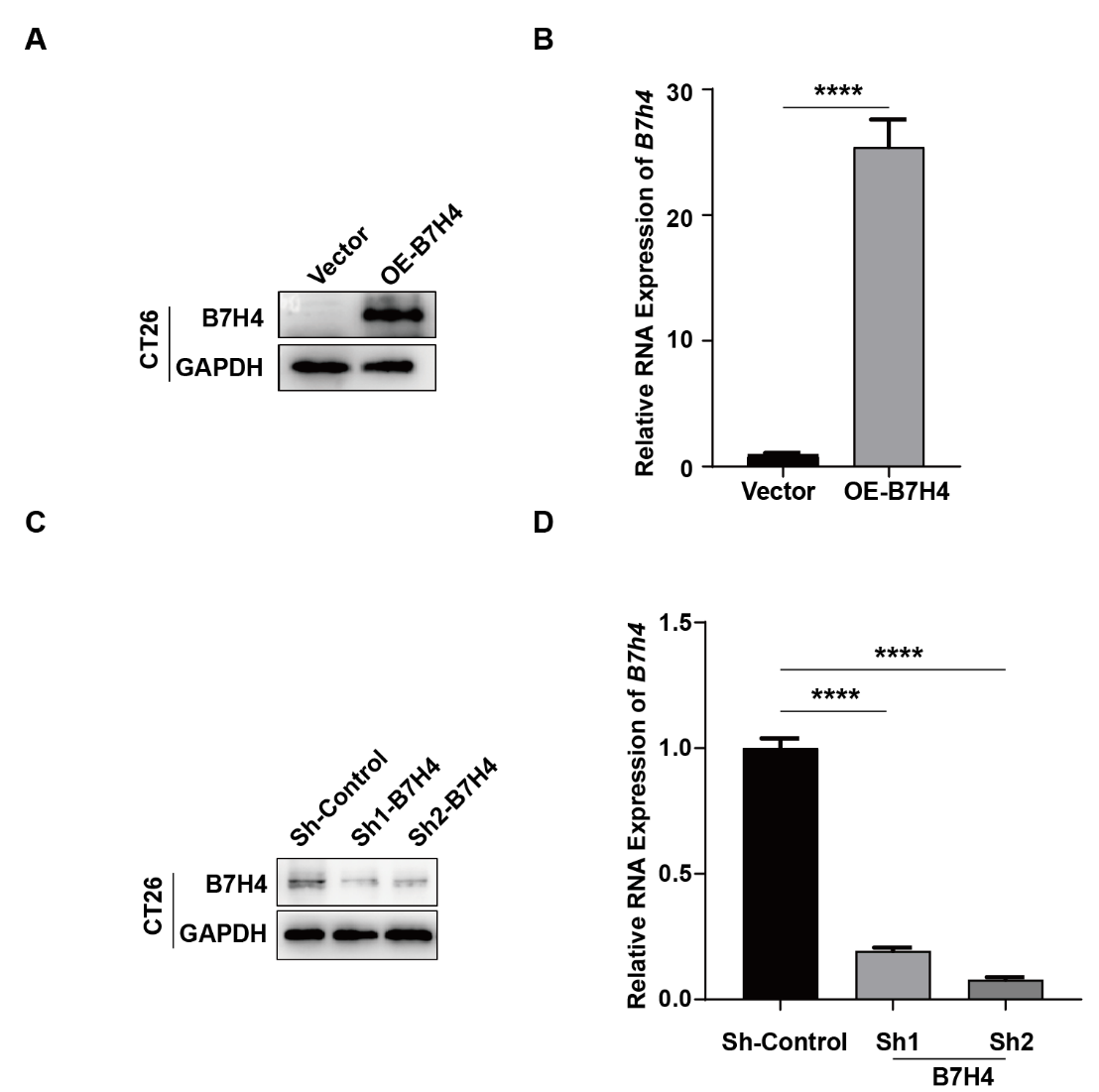


**Supplementary Fig. 1 Verification of stable strain of CT26-B7H4 or CT26-shB7H4**

**A** The expression of B7H4 protein in vector group and OE-B7H4 group was detected by Western Blot.

**B** The relative RNA expression of *B7h4* in vector group and OE-B7H4 group was detected by qPCR. Student’s *t* test (****, P < 0.0001).

**C** The expression of B7H4 protein in control group and sh1-B7H4sh2-B7H4 group was detected by Western Blot.

**D** The relative RNA expression of *B7h4* in control group and sh1-B7H4sh2-B7H4 group was detected by qPCR. Student’s *t* test (****, P < 0.0001).

**
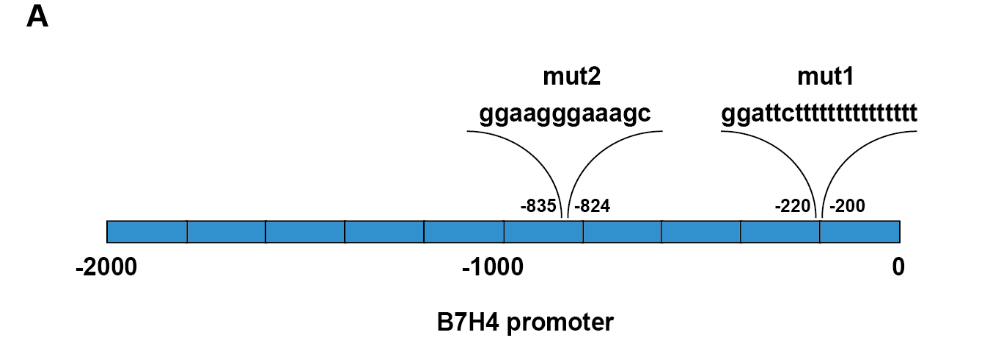
**

**Supplementary Fig. 2 Sequence of B7H4 promoter region and possible binding sites to IRF1**

**The original Western blotting images of Fig. 2c in the text is as follows:**

**
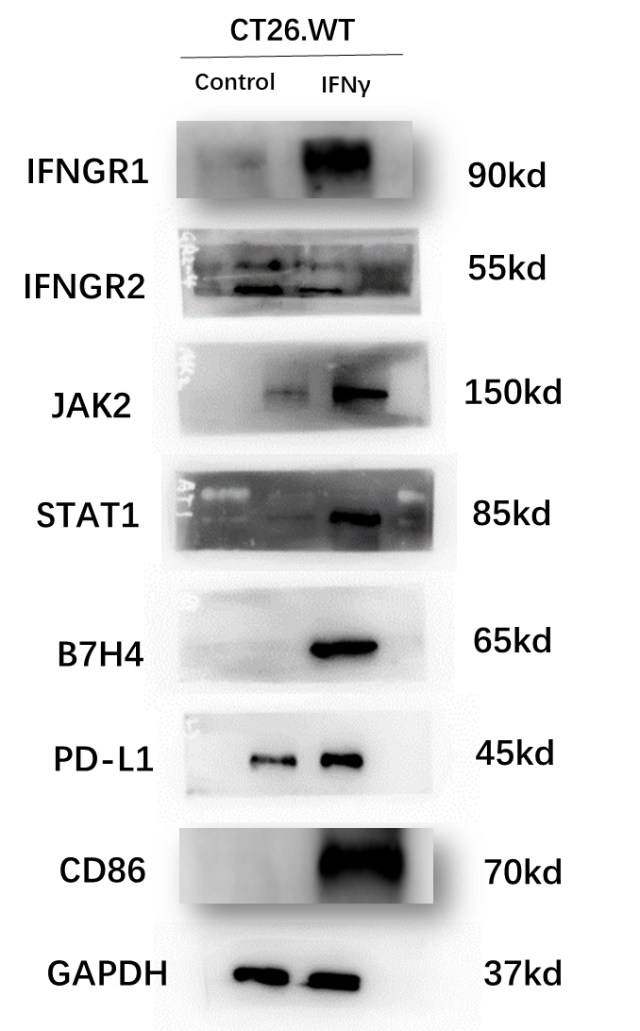
**

**Supplementary Fig. 3** The expression of interferon signaling pathway proteins in CT26.WT cell line was measured by Western blotting before and after IFN-γ stimulation.

**
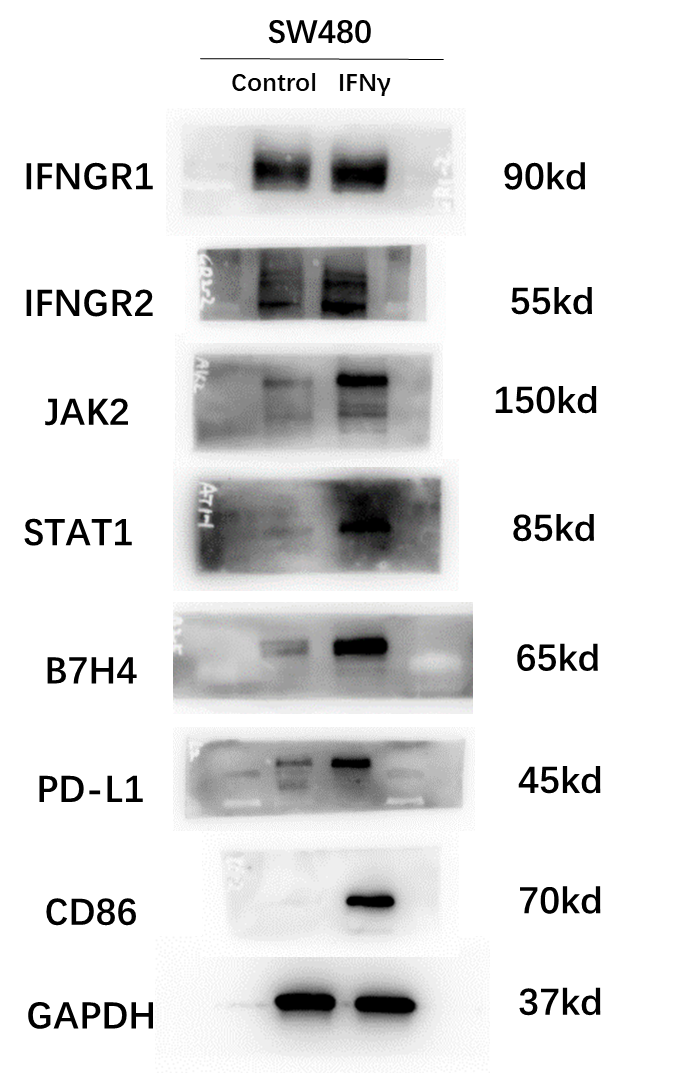
**

**Supplementary Fig. 4** The expression of interferon signaling pathway proteins in SW480 cell line was measured by Western blotting before and after IFN-γ stimulation.

**
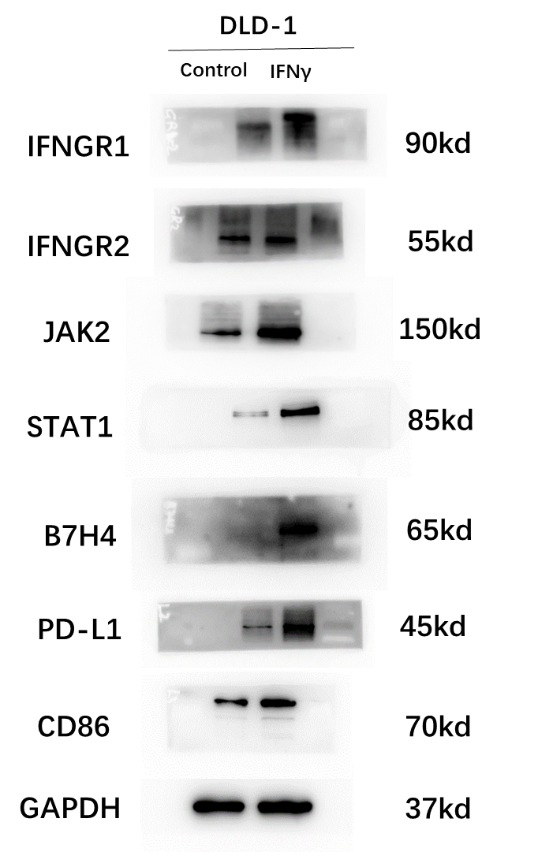
**

**Supplementary Fig.5** The expression of interferon signaling pathway proteins in DLD-1 cell line was measured by Western blotting before and after IFN-γ stimulation.

**
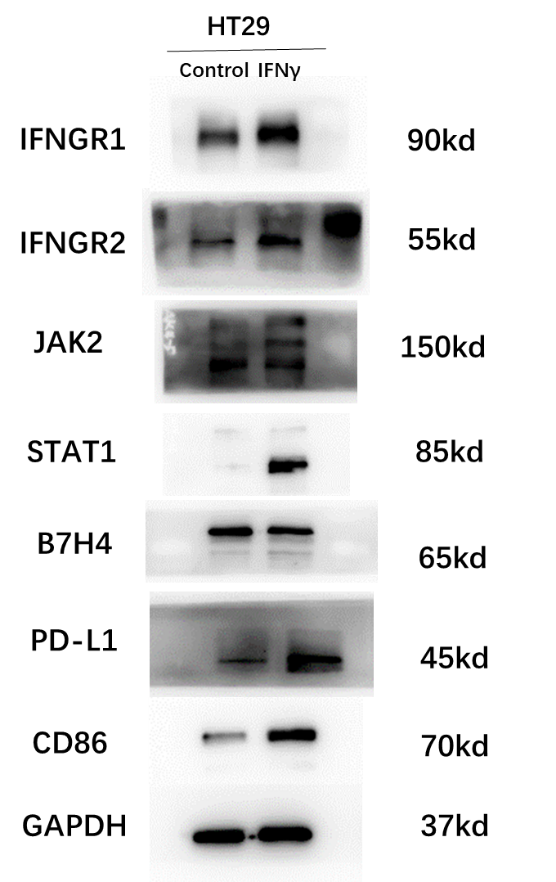
**

**Supplementary Fig.6** The expression of interferon signaling pathway proteins in HT29 cell line was measured by Western blotting before and after IFN-γ stimulation.

**
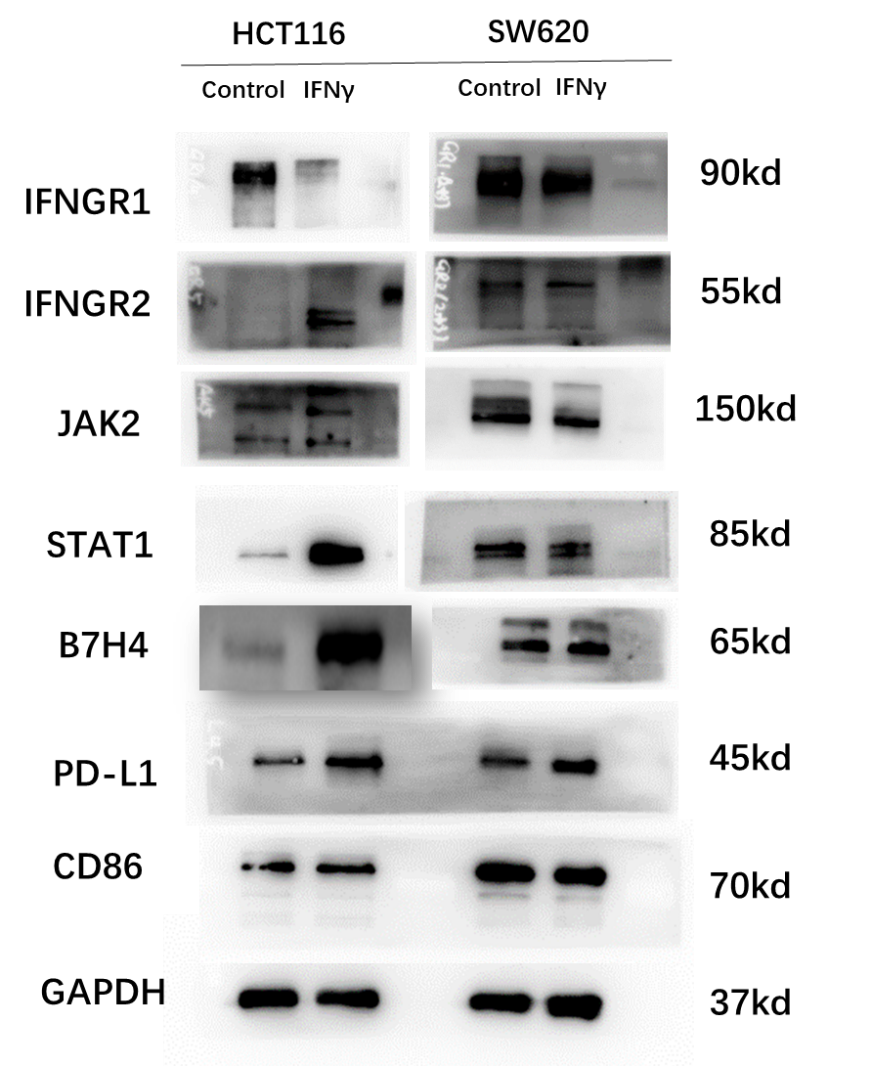
**

**Supplementary Fig.7** The expression of interferon signaling pathway proteins in HCT116 and SW620 cell lines was measured by Western blotting before and after IFN-γ stimulation.

**
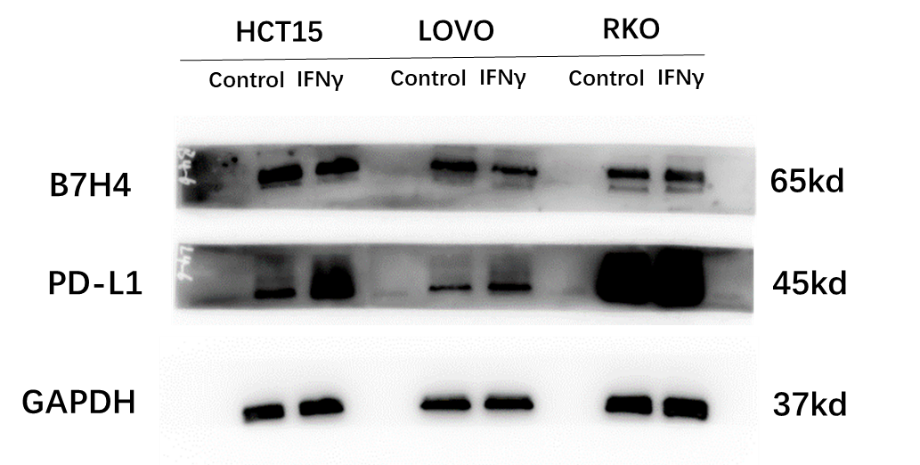
**

**Supplementary Fig.8** The expression of B7H4 and PD-L1 in HCT15 、LOVO and RKO cell lines was measured by Western blotting before and after IFN-γ stimulation.

**
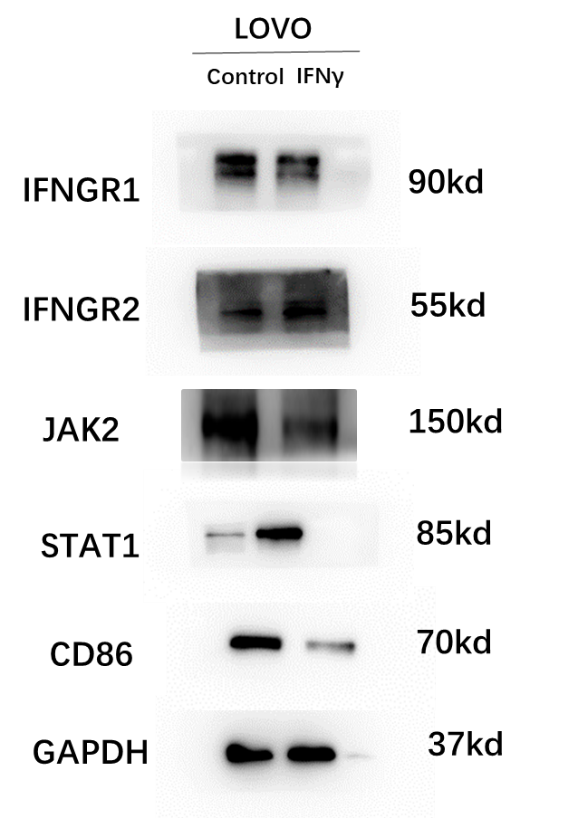
**

**Supplementary Fig.9** The expression of interferon signaling pathway proteins in LOVO cell line was measured by Western blotting before and after IFN-γ stimulation.

**The original Western blotting images of Fig. 5a in the text is as follows:**


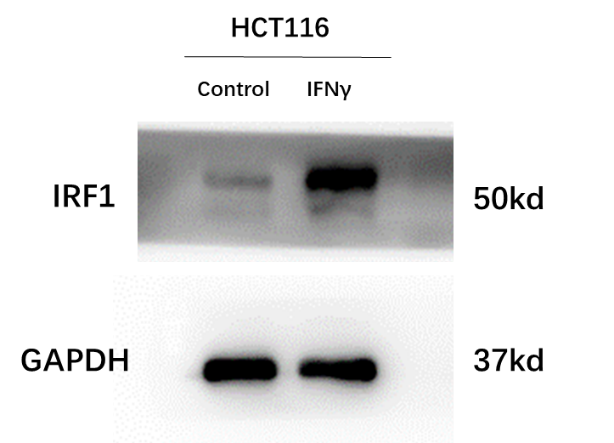


**Supplementary Fig.10** The expression of IRF1 in HCT116 cell before and after IFN-γ stimulation was determined by Western blotting.


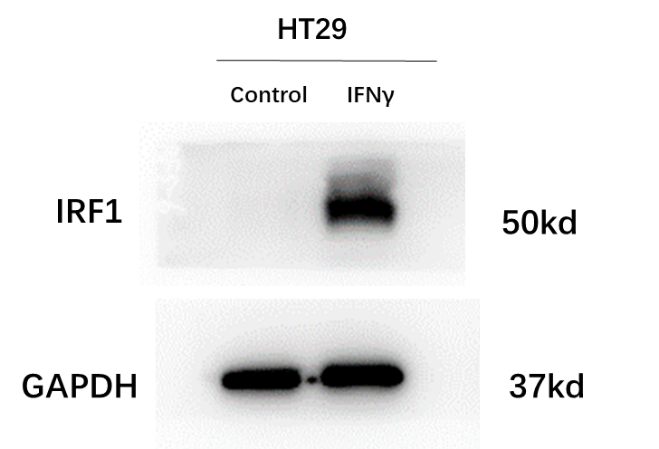


**Supplementary Fig.11** The expression of IRF1 in HT29 cell before and after IFN-γ stimulation was determined by Western blotting.


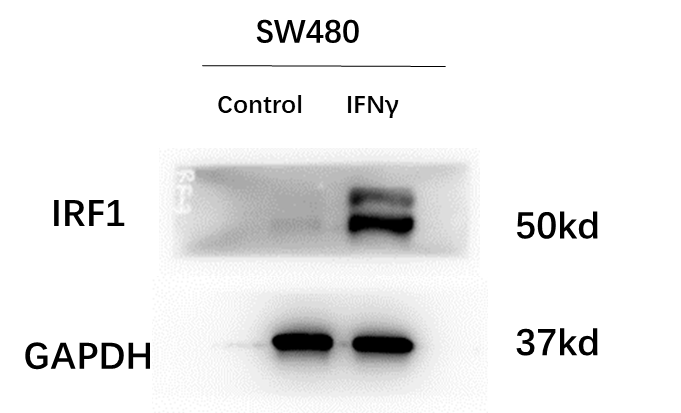


**Supplementary Fig.12** The expression of IRF1 in SW480 cell before and after IFN-γ stimulation was determined by Western blotting.


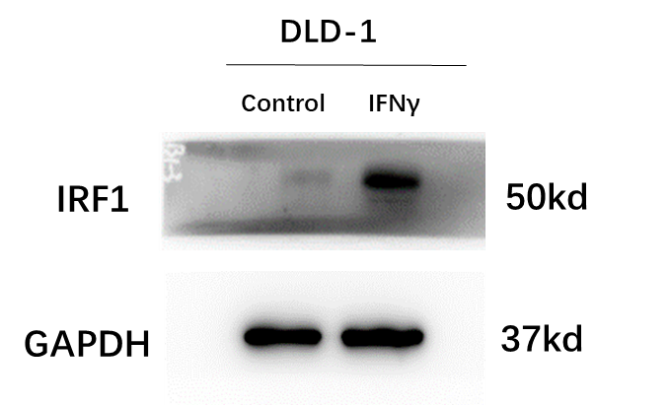


**Supplementary Fig.13** The expression of IRF1 in DLD-1 cell before and after IFN-γ stimulation was determined by Western blotting.


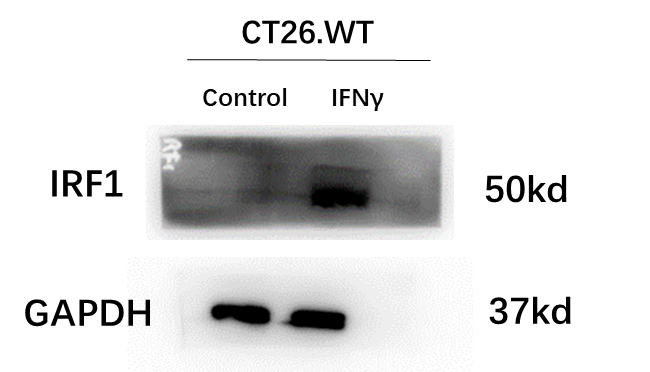


**Supplementary Fig.14** The expression of IRF1 in CT26.WT cell before and after IFN-γ stimulation was determined by Western blotting.

**The original Western blotting images of Fig. 5g in the text is as follow:**


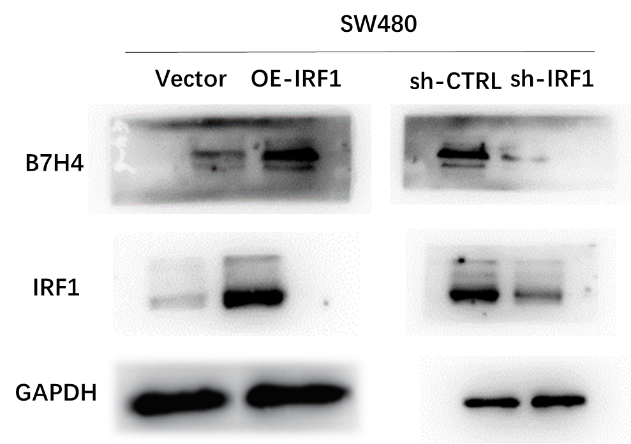


**Supplementary Fig.15** Changes in B7H4 expression after overexpression of IRF1 (OE-IRF1) or silencing of IRF1 (sh-IRF1) were evaluated by Western blotting in SW480.

**The original Western blotting images of Fig.5h in the text is as follow:**

**
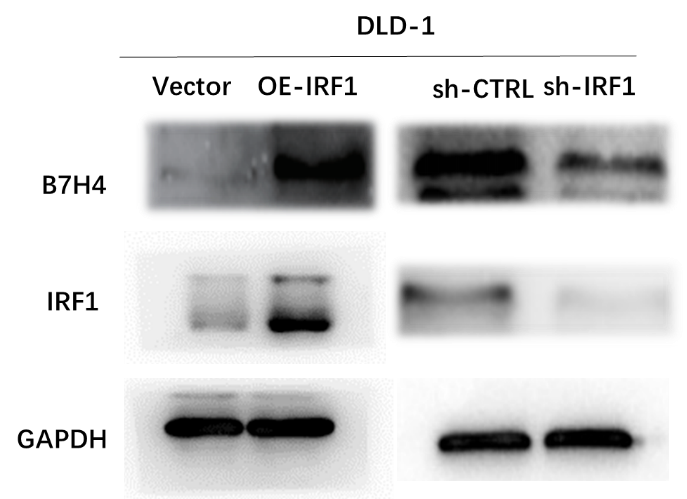
**

**Supplementary Fig.16** Changes in B7H4 expression after overexpression of IRF1 (OE-IRF1) or silencing of IRF1 (sh-IRF1) were evaluated by Western blotting in DLD-1.

**The structure of pGL4.10-B7H4 promoter mut1, and pGL4.10-B7H4 promoter mut2 vectors：**

**pGL4.10-B7H4 promoter wt vector construction**

1. Gene name: B7H4 promoter wt
2. Sequence length: 2012bp
3. Carrier name: pgl4.10
4. Cloning site: KpnI--XhoI (GGTACC/CTCGAG)
5. Construction sequence:

TTGAGGTGGGGCTGCTTTTTTGTTTTTTTTTTAAGACAGCATCTTGCTTTGTTGCCCATCGTGGAGTATACTGGCACGATCATGGCTCACTGCAGCTTCAAGCCCCACTCAAGTGATCCTCCCACCCCAGCCTCCTGAGCAGCTGGGACTATATGCCCAGCTAATTTTTTGTTTGTTTTCCTGTTACTAGATGGATTCTTTTTTTTTTTTTTTAAATTTTGTAGAGACGAAGTCTCAGTATGCTGCTCAGGCTAGTTTCGAACTGCTAGGCTCAAGAATTCCTCCTACCAGCCAGGCGCAGTGGCTCATGCCTGTAATTCCAGTACTTGGGGAAGTCGAGGTGGGTGGATCACTTGAGGTTGGGAGTTCGAGACCAGCTTGGCCAATATGGAGAAACCCCGTCTCTACTAAAAATACAAAAATTAGCTGAGTGCGGTGGTGCACCTATAATCCCATCTACTTGGGATGCTGAGGCAGGAGAATCGCTTGAACCCAGGAGGCAGAGGTTACAGTTACAGAGCCAAGATTGTACCATTGCACTCCAGCCTGGACAACAGAGCGAGACTCAGTCTCAAAAAAAAAAAATTCCTCCTACCTTGGCCTCCCAAAATATTGGGATTATAAGCTTGAGCCACTGTGCCCAGCTGAAGCTGCTCTTCTTAAGGCTCATTGAGGGGTAGGGCAGAATCCCTGTGCCTTTGGTTGGAATTACAGAAACCACTGCTCCCACTGCCCTCAGCTTCCCCAGACCCGGGAAGTGACCATTCAGCACTGGCACTGCATAGATTCCAGGGCATCACCCTTTACTTATTCTGGGGAAGGGAAAGCACATAGACCCGCCTCTCATTTATCCCTCACTGCAACCCAGTGAGACACACATTGAAGTAAAGTGATGTTCTCAAAGCCACATGATTAATTAGGAACCCAGGAATTGAACCTAGCTGTTTGACTCTAAAGGTCTTTCCACTGTGACCTTGCCCTCTCCTGCTGCCTTATTTTCACCACTCAGTTCTCTCTGCTCCATCTTTACTCAATGGTTGCTCCACCTGCCCATACGCAAACACTAGGAGGATGCTGCTGAAGGCCTGGAGCACCTTCACTGCAAACATAGTCAGTCAGCAGCAGCAGTAAGCCAGGCAACCAGTCATGAAACTTTCCCATCCAAGGTACTTCTGGGGGCTGGAGGAAACAATAAGGAGAGAGAGAGAGAATAAGAGAGAGAGAGAGCAACGTTTAGGCTTTAACGAGAATCTTGCCTAGTTGGCAATCTGCTGGATGAAATGTCAGAGATTTTTGCTAACCCACTCACCTTTGACAGCAATAGCTTCTGAGTATTGGGATCTCCAGAGAGAGGGGCAGTTGAAGCAGAGCACATTCAGTGTCTTTTTGGAGTCTCAAAAAAATTGCAAGGTGGGAAGTGCTGGCCCTCTCCTGCTCCCACTGAAATGAAGCTGGAAGAGAATTGCACACCCAGCTGGGCATCTCAGGAGACCTAGGCAAGTTCAGTCTTGAGTGACAAACTCAGTGGTCTTGTCACTTCAGCTCATGGCCTTCCTGAGGTCCCCCAGCATGAGTCTCCTCCACGAGAGACCTGACCAATGGTGACCCAGCCTCTGTTTGAGCGTCATCTACCAGCAGAGATGGGGACTCCTGGCCCCAAGGCTCCCAGCATAGCCTGACACTTTGAGCTATTCCAGTCCTTTCTTATTTTGAGATTAAATCTCCCTCCCTACAGCTCCCTTCCACAGTCAGGAGGAAAGTCCTGCTGAATGCAGGCCCTGAAGCAAGGGCCAGGGAAGCTTTTCCTCGTCACCCTCCCAAAGTCAAGACTTGGAAAGGCAGCTCTGAGCCCCTGGCTTGGCTGGCTGGCCGGAGCAGGCAGCCACTGTGCCTGCAGGGAATTCTGCACAGCCAGTTTCCTCATACCTGAGCCGTCTACAGCTGCACGCACCACTCCCGGCCTCAACACACTATTTAAGGCCAATACACGGGAGCTGGTT

**Construction of pGL4.10-B7H4 promoter mut1 vector**

1. Gene name: B7H4 promoter mut1 (removing yellow background site)
2. Sequence length: 1991bp
3. Carrier name: pgl4.10
4. Cloning site: KpnI--XhoI （GGTACC/CTCGAG）
5. Construction sequence:

TTGAGGTGGGGCTGCTTTTTTGTTTTTTTTTTAAGACAGCATCTTGCTTTGTTGCCCATCGTGGAGTATACTGGCACGATCATGGCTCACTGCAGCTTCAAGCCCCACTCAAGTGATCCTCCCACCCCAGCCTCCTGAGCAGCTGGGACTATATGCCCAGCTAATTTTTTGTTTGTTTTCCTGTTACTAGATAAATTTTGTAGAGACGAAGTCTCAGTATGCTGCTCAGGCTAGTTTCGAACTGCTAGGCTCAAGAATTCCTCCTACCAGCCAGGCGCAGTGGCTCATGCCTGTAATTCCAGTACTTGGGGAAGTCGAGGTGGGTGGATCACTTGAGGTTGGGAGTTCGAGACCAGCTTGGCCAATATGGAGAAACCCCGTCTCTACTAAAAATACAAAAATTAGCTGAGTGCGGTGGTGCACCTATAATCCCATCTACTTGGGATGCTGAGGCAGGAGAATCGCTTGAACCCAGGAGGCAGAGGTTACAGTTACAGAGCCAAGATTGTACCATTGCACTCCAGCCTGGACAACAGAGCGAGACTCAGTCTCAAAAAAAAAAAATTCCTCCTACCTTGGCCTCCCAAAATATTGGGATTATAAGCTTGAGCCACTGTGCCCAGCTGAAGCTGCTCTTCTTAAGGCTCATTGAGGGGTAGGGCAGAATCCCTGTGCCTTTGGTTGGAATTACAGAAACCACTGCTCCCACTGCCCTCAGCTTCCCCAGACCCGGGAAGTGACCATTCAGCACTGGCACTGCATAGATTCCAGGGCATCACCCTTTACTTATTCTGGGGAAGGGAAAGCACATAGACCCGCCTCTCATTTATCCCTCACTGCAACCCAGTGAGACACACATTGAAGTAAAGTGATGTTCTCAAAGCCACATGATTAATTAGGAACCCAGGAATTGAACCTAGCTGTTTGACTCTAAAGGTCTTTCCACTGTGACCTTGCCCTCTCCTGCTGCCTTATTTTCACCACTCAGTTCTCTCTGCTCCATCTTTACTCAATGGTTGCTCCACCTGCCCATACGCAAACACTAGGAGGATGCTGCTGAAGGCCTGGAGCACCTTCACTGCAAACATAGTCAGTCAGCAGCAGCAGTAAGCCAGGCAACCAGTCATGAAACTTTCCCATCCAAGGTACTTCTGGGGGCTGGAGGAAACAATAAGGAGAGAGAGAGAGAATAAGAGAGAGAGAGAGCAACGTTTAGGCTTTAACGAGAATCTTGCCTAGTTGGCAATCTGCTGGATGAAATGTCAGAGATTTTTGCTAACCCACTCACCTTTGACAGCAATAGCTTCTGAGTATTGGGATCTCCAGAGAGAGGGGCAGTTGAAGCAGAGCACATTCAGTGTCTTTTTGGAGTCTCAAAAAAATTGCAAGGTGGGAAGTGCTGGCCCTCTCCTGCTCCCACTGAAATGAAGCTGGAAGAGAATTGCACACCCAGCTGGGCATCTCAGGAGACCTAGGCAAGTTCAGTCTTGAGTGACAAACTCAGTGGTCTTGTCACTTCAGCTCATGGCCTTCCTGAGGTCCCCCAGCATGAGTCTCCTCCACGAGAGACCTGACCAATGGTGACCCAGCCTCTGTTTGAGCGTCATCTACCAGCAGAGATGGGGACTCCTGGCCCCAAGGCTCCCAGCATAGCCTGACACTTTGAGCTATTCCAGTCCTTTCTTATTTTGAGATTAAATCTCCCTCCCTACAGCTCCCTTCCACAGTCAGGAGGAAAGTCCTGCTGAATGCAGGCCCTGAAGCAAGGGCCAGGGAAGCTTTTCCTCGTCACCCTCCCAAAGTCAAGACTTGGAAAGGCAGCTCTGAGCCCCTGGCTTGGCTGGCTGGCCGGAGCAGGCAGCCACTGTGCCTGCAGGGAATTCTGCACAGCCAGTTTCCTCATACCTGAGCCGTCTACAGCTGCACGCACCACTCCCGGCCTCAACACACTATTTAAGGCCAATACACGGGAGCTGGTT

**Construction of pGL4.10-B7H4 promoter mut2 vector**

1. Gene name: B7H4 promoter mut2 (Remove green background dots)
2. Sequence length: 2000bp
3. Carrier name: pgl4.10
4. Cloning site: KpnI--XhoI （GGTACC/CTCGAG）
5. Construction sequence:

TTGAGGTGGGGCTGCTTTTTTGTTTTTTTTTTAAGACAGCATCTTGCTTTGTTGCCCATCGTGGAGTATACTGGCACGATCATGGCTCACTGCAGCTTCAAGCCCCACTCAAGTGATCCTCCCACCCCAGCCTCCTGAGCAGCTGGGACTATATGCCCAGCTAATTTTTTGTTTGTTTTCCTGTTACTAGATGGATTCTTTTTTTTTTTTTTTAAATTTTGTAGAGACGAAGTCTCAGTATGCTGCTCAGGCTAGTTTCGAACTGCTAGGCTCAAGAATTCCTCCTACCAGCCAGGCGCAGTGGCTCATGCCTGTAATTCCAGTACTTGGGGAAGTCGAGGTGGGTGGATCACTTGAGGTTGGGAGTTCGAGACCAGCTTGGCCAATATGGAGAAACCCCGTCTCTACTAAAAATACAAAAATTAGCTGAGTGCGGTGGTGCACCTATAATCCCATCTACTTGGGATGCTGAGGCAGGAGAATCGCTTGAACCCAGGAGGCAGAGGTTACAGTTACAGAGCCAAGATTGTACCATTGCACTCCAGCCTGGACAACAGAGCGAGACTCAGTCTCAAAAAAAAAAAATTCCTCCTACCTTGGCCTCCCAAAATATTGGGATTATAAGCTTGAGCCACTGTGCCCAGCTGAAGCTGCTCTTCTTAAGGCTCATTGAGGGGTAGGGCAGAATCCCTGTGCCTTTGGTTGGAATTACAGAAACCACTGCTCCCACTGCCCTCAGCTTCCCCAGACCCGGGAAGTGACCATTCAGCACTGGCACTGCATAGATTCCAGGGCATCACCCTTTACTTATTCTGGACATAGACCCGCCTCTCATTTATCCCTCACTGCAACCCAGTGAGACACACATTGAAGTAAAGTGATGTTCTCAAAGCCACATGATTAATTAGGAACCCAGGAATTGAACCTAGCTGTTTGACTCTAAAGGTCTTTCCACTGTGACCTTGCCCTCTCCTGCTGCCTTATTTTCACCACTCAGTTCTCTCTGCTCCATCTTTACTCAATGGTTGCTCCACCTGCCCATACGCAAACACTAGGAGGATGCTGCTGAAGGCCTGGAGCACCTTCACTGCAAACATAGTCAGTCAGCAGCAGCAGTAAGCCAGGCAACCAGTCATGAAACTTTCCCATCCAAGGTACTTCTGGGGGCTGGAGGAAACAATAAGGAGAGAGAGAGAGAATAAGAGAGAGAGAGAGCAACGTTTAGGCTTTAACGAGAATCTTGCCTAGTTGGCAATCTGCTGGATGAAATGTCAGAGATTTTTGCTAACCCACTCACCTTTGACAGCAATAGCTTCTGAGTATTGGGATCTCCAGAGAGAGGGGCAGTTGAAGCAGAGCACATTCAGTGTCTTTTTGGAGTCTCAAAAAAATTGCAAGGTGGGAAGTGCTGGCCCTCTCCTGCTCCCACTGAAATGAAGCTGGAAGAGAATTGCACACCCAGCTGGGCATCTCAGGAGACCTAGGCAAGTTCAGTCTTGAGTGACAAACTCAGTGGTCTTGTCACTTCAGCTCATGGCCTTCCTGAGGTCCCCCAGCATGAGTCTCCTCCACGAGAGACCTGACCAATGGTGACCCAGCCTCTGTTTGAGCGTCATCTACCAGCAGAGATGGGGACTCCTGGCCCCAAGGCTCCCAGCATAGCCTGACACTTTGAGCTATTCCAGTCCTTTCTTATTTTGAGATTAAATCTCCCTCCCTACAGCTCCCTTCCACAGTCAGGAGGAAAGTCCTGCTGAATGCAGGCCCTGAAGCAAGGGCCAGGGAAGCTTTTCCTCGTCACCCTCCCAAAGTCAAGACTTGGAAAGGCAGCTCTGAGCCCCTGGCTTGGCTGGCTGGCCGGAGCAGGCAGCCACTGTGCCTGCAGGGAATTCTGCACAGCCAGTTTCCTCATACCTGAGCCGTCTACAGCTGCACGCACCACTCCCGGCCTCAACACACTATTTAAGGCCAATACACGGGAGCTGGTT
